# Linking behaviour change techniques to mechanisms of action: Using the Theory and Techniques Tool alongside the Behaviour Change Intervention Ontology

**DOI:** 10.12688/wellcomeopenres.23879.1

**Published:** 2025-04-11

**Authors:** Lisa Zhang, Paulina M. Schenk, Micaela Santilli, Alison J Wright, Marta M. Marques, Marie Johnston, Robert West, Susan Michie

**Affiliations:** 1 Centre for Behaviour Change, University College London, London, England, UK; 2 Institute of Pharmaceutical Science, King's College London, London, England, UK; 3 NOVA National School of Public Health, Comprehensive Health Research Centre (CHRC), NOVA University of Lisbon, Lisbon, Portugal; 4 Aberdeen Health Psychology Group, University of Aberdeen, Aberdeen, Scotland, UK

**Keywords:** behaviour change; intervention; ontology; theory; mechanisms of action; behaviour change techniques

## Abstract

**Background:**

Understanding how interventions work requires clear hypotheses, rigorous testing, and accurate reporting of links between behaviour change techniques (BCTs)—the smallest replicable active components of interventions—and mechanisms of action (MoAs), the processes through which behaviour changes. The Theory and Technique Tool (TaTT) provides a grid of likely BCT-MoA links to guide intervention design, based on literature synthesis and expert consensus. Recently, the Behaviour Change Intervention Ontology development team introduced detailed, computer-readable lower-level ontologies for BCTs and MoAs, but limited guidance exists on integrating the BCT-MoA links proposed by the TaTT with these ontologies. This study aimed to map BCTs and MoAs from the TaTT to corresponding classes (i.e., categorisations or groupings) in the Behaviour Change Technique Ontology (BCTO) and Mechanism of Action (MoA) Ontology.

**Methods:**

Three researchers mapped the classes from the BCTO onto 74 BCTs within the TaTT, using their definitions. Similarly, two researchers mapped classes from the MoA Ontology onto the 26 MoAs within the TaTT. Discrepancies were resolved through discussion with senior researchers. Subsequent updates to the BCT and MoA Ontologies necessitated a researcher updating the mappings, with the revisions being verified by the research team.

**Results:**

From the BCTO, 85 BCTs were mapped to the 74 BCTs present in the TaTT, while 56 MoAs from the MoA Ontology were mapped to the 26 MoAs present in the TaTT. Subclasses of these 85 BCTs and 56 MoAs provide additional specificity and can be found by further engaging with these ontologies.

**Discussion:**

Mapping the TaTT to the Behaviour Change Intervention Ontology enhances clarity and precision in selecting and reporting BCT-MoA links, enabling integration of data across frameworks. Future work should maintain these mappings as ontologies evolve and users provide more feedback and evidence on BCTs, MoAs, and their links, ensuring they remain relevant and user-friendly.

## Plain language summary

Understanding how interventions change people’s behaviours is important for making them more effective. Behaviour change interventions include specific actions called “behaviour change techniques” (“BCTs”), such as encouraging people to set goals for their behaviour. These BCTs are the “active ingredients” of interventions that can be observed and repeated. BCTs work by affecting processes called mechanisms of action (MoAs), i.e. how the intervention leads to change. For example, MoAs might involve changing a person’s beliefs, improving their abilities, or increasing their access to resources.

To support intervention developers in selecting BCTs to target specific MoAs, an online resource, called the Theory and Technique Tool (TaTT), was previously developed. This tool provides an evidence-based grid showing which BCTs are likely or unlikely to change certain MoAs. Recently, new tools—the Behaviour Change Technique Ontology (BCTO) and Mechanisms of Action Ontology (MoA Ontology)—were developed to include a wider range of BCTs and MoAs and provide more precise and computer-readable BCT and MoA definitions. By aligning the TaTT with these newer tools, we can support (1) ontology users in hypothesising about likely BCT-MoA links, and (2) TaTT users in identifying more detailed yet relevant BCTs and MoAs from the ontologies and using these in computer applications.

This study aimed to map the newer ontologies’ categories to the TaTT’s 74 BCTs and 26 MoAs. Researchers carefully compared and discussed definitions from both tools to create mappings. The study found that 85 BCTs in the newer ontology corresponded to 74 BCTs from the TaTT, and 56 MoAs in the newer ontology corresponded to 26 MoAs from the TaTT.

By linking the ontologies to the TaTT, this work makes it easier to use these tools together. This helps design and report behaviour change interventions more clearly and supports advanced uses like automated data analysis.

## Table of acronyms

**Table:** 

Acronym	Meaning
BCIO	Behaviour Change Intervention Ontology
BCIOSearch	Behaviour Change Intervention Ontology Search Tool
BCT	Behaviour Change Technique
BCTO	Behaviour Change Technique Ontology
BCTTv1	Behaviour Change Technique Taxonomy V1
HBCP	Human Behaviour Change Project
MoA	Mechanisms of Action
TaTT	Theory and Techniques Tool

## Introduction

Behaviour change interventions have the potential to address critical policy areas, such as health and sustainability, by influencing relevant behaviours ( Albarracín *et al*., 2024; Michie & West, 2013; Newell *et al*., 2021). However, these interventions often show mixed effectiveness at changing target behaviours ( Jepson *et al*., 2010; Johnson & May, 2015). To improve intervention effectiveness, established guidance on developing and evaluating interventions, such as the UK Medical Research Council Framework, advocate the use of ***theory*** to inform interventions ( Craig *et al*., 2008; Skivington *et al*., 2021) (see glossary of bold, italicised terms in Table 1). Theories have various roles in supporting intervention design, which include helping:

- identify ***mechanisms of action (MoAs***; the processes through which interventions bring about their influence on behaviour) to understand causal processes behind interventions ( Michie *et al*., 2008)
- identify important, relevant and feasible outcomes that an intervention intends to target ( Davidoff *et al*., 2015)
- inform the content and delivery of an intervention ( O’Cathain *et al*., 2019)
- reduce research waste by summarising the current state of knowledge, providing a framework to falsify incorrect assumptions and facilitate accumulation of evidence, and guiding future research ( Davidoff *et al*., 2015; Gardner *et al*., 2010; Lippke & Ziegelmann, 2008)

**Table 1.** Glossary of terms ( Marques *et al*., 2024b; Michie *et al*., 2017; Schenk *et al*., 2024b).

Term	Definition	Source
Behaviour change technique	A planned process that is the smallest part of BCI content that is observable, replicable and on its own has the potential to bring about behaviour change.	( Marques *et al*., 2024b)
Behaviour Change Technique Ontology	A lower-level ontology of the Behaviour Change Intervention Ontology, which includes classes for BCTs, with clear labels, definitions and computer-readable alphanumeric IDs (URIs), and specifies relationships between these classes.	( Marques *et al*., 2024b)
Class	Classes in ontologies represent types of entities in the world. The terms “entity” and “class” can be used interchangeably to refer to the entities represented in an ontology. Classes can be arranged hierarchically by the specification of parent and child classes; see definition of parent class in the glossary	Arp *et al*. (2015)
Entity	Anything that exists, including objects, processes, and their attributes.	Arp *et al*. (2015)
GitHub	A web-based platform used as a repository for sharing code, allowing version control.	https://github.com/
Mechanism of action	A process that is causally active in the relationship between a Behaviour Change Intervention scenario and its outcome behaviour.	Schenk *et al*. (2024b)
Mechanism of Action Ontology	A lower-level ontology of the Behaviour Change Intervention Ontology, which includes classes for MoAs, with clear labels, definitions and computer-readable alphanumeric IDs (URIs), and specifies relationships between these classes.	Schenk *et al*. (2024b)
Ontology	A standardised representational framework providing a set of classes for the consistent description (or “annotation” or “tagging”) of data and information across disciplinary and research community boundaries.	Arp *et al*., (2015)
Parent class	A class within an ontology that is hierarchically related to one or more child classes (subclasses) such that all members of the child class are also members of the parent class, and all properties of the parent class are also properties of the child class.	Arp *et al*., (2015)
Relationship	The manner in which two classes are connected or linked.	Arp *et al*., (2015)
Lower-level ontology	A part of a broader ontology, which captures classes and relationships that fall within a specific discrete scope. Also referred to as “lower-level ontology”.	Sari *et al*. (2013)
Theory	A set of constructs and/or statements that describe, explain and predict phenomena.	Davis *et al*. (2015)
Theoretical construct	A concept proposed within a theory.	Michie *et al*. (2005)
Theory and Technique Tool	An online interactive tool that includes an evidence-based grid of ‘likely’ links between BCTs and MoAs.	https://theoryandtechniquetool.humanbehaviourchange.org/
URI	A string of characters that unambiguously identifies an ontology or an individual entity within an ontology. Having URI identifiers is one of the OBO Foundry principles.	http://www.obofoundry.org/principles/fp-003-uris.html

**Figure 1.**
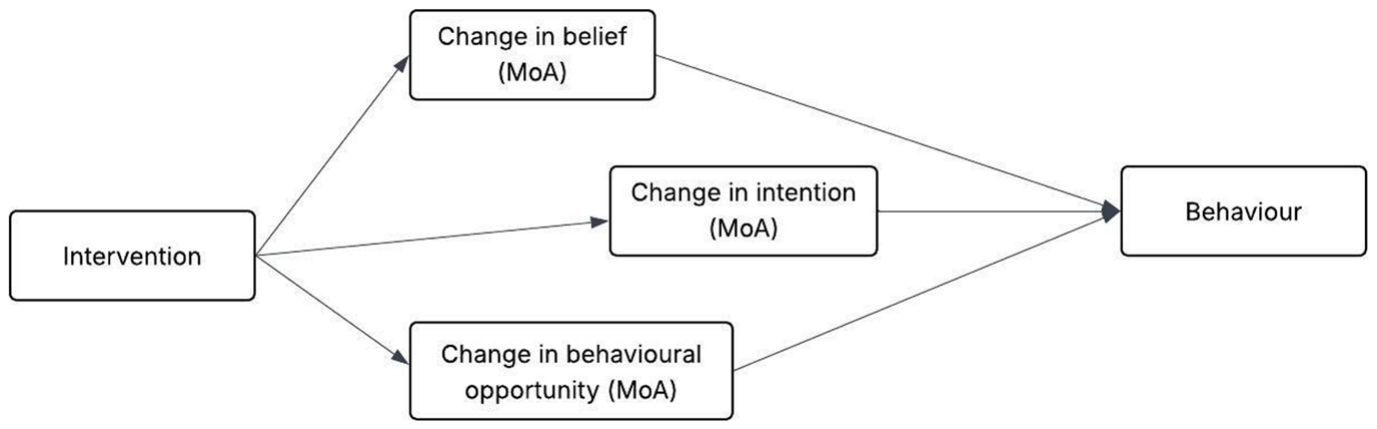
Representation of an example link between intervention, its MoAs and target behaviour. This figure has been reproduced with permission from Schenk *et al*. (2024b).

Specifically, by supporting intervention designers to select, target and test their interventions’ MoAs (e.g., motivation, capability or opportunity), theories support our understanding of how interventions work and thereby can inform future intervention designs ( Carey *et al*., 2019; Michie *et al*., 2018; Schenk *et al*., 2024b). For example, various theories propose ‘self-efficacy belief’ to be an important ***theoretical construct*** for changing behaviours ( Bandura, 1997; Locke & Latham, 1990; Luszczynska & Schwarzer, 2015; Rosenstock, 1974), and based on this, intervention designers can hypothesise self-efficacy as an MoA in their own intervention and test or further explore it. A representation of how interventions work through MoAs to change behaviours is shown in Figure 1. As part of interventions, ***behaviour change techniques (BCTs)*** can be used to target MoAs. BCTs have been defined as “ *a part of the content of a behaviour change intervention that are observable, replicable and on their own have the potential to bring about behaviour change*” ( Marques *et al*., 2024b, p., 8). An example of a link between a BCT and MoA would be: Altering a participants’ environment (BCT) which changes the participants’ opportunities (MoA) to enact a behaviour.

Despite calls for increased and better use of theories, many intervention reports lack explicit and clear descriptions of how theories were used during intervention development and evaluation ( Dalgetty *et al*., 2019; Mama *et al*., 2015; Prestwich *et al*., 2014; Prestwich *et al*., 2015). This includes poor reporting of the links between intervention components (e.g., BCTs) and specific theoretical constructs (including potential MoAs). For example, a meta-analysis found that only half the included studies explicitly reported a theory base, and of these, 90% did not report links between the BCTs used with specific theoretical constructs ( Prestwich *et al*., 2014). This lack of reporting may, in part, stem from researchers having to navigate an increasingly complex theoretical landscape, with over 80 behavioural theories, many of which do not explicitly link BCTs to potential MoAs ( Davis *et al*., 2015; Michie *et al*., 2008).

### The Theory and Techniques Tool

**Figure 2.**
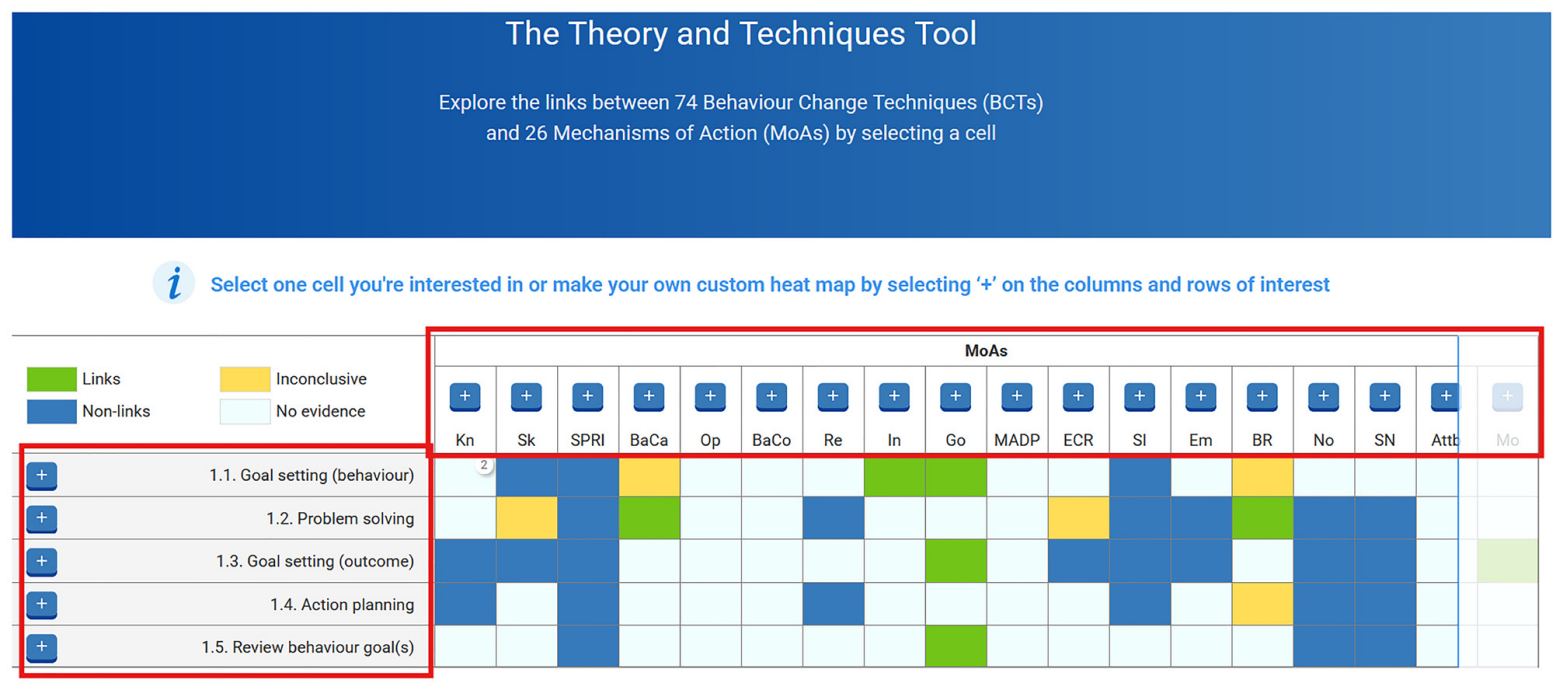
Screenshot of the Theory and Technique Tool (TaTT).

To provide practical guidance on selecting BCTs to target MoAs in interventions, an online evidence-based grid that shows ‘likely’ BCT-MoA links ^1^, the ***Theory and Technique Tool (TaTT)**,* was developed. These links were between 74 BCTs selected from the 93 BCTs of the Behaviour Change Techniques Taxonomy v1 (BCTTv1; Michie *et al*., 2013) and 26 MoAs. The 74 BCTs were the most commonly occurring ones, from the 93 BCTs, in a literature review ( Carey *et al*., 2019). The 26 MoAs included 14 MoAs from the Theoretical Domains Framework ( Cane *et al*., 2012) and 12 frequently occurring MoAs ^2^ identified from 83 behaviour change theories ( Davis *et al*., 2015). Figure 2 shows a screenshot of the TaTT, with the red box on the left-hand side showing some of the 74 BCTs, and the horizontal red box showing the abbreviated labels of some of the 26 MoAs in this tool. The labels and definitions for these 74 BCTs and 26 MoAs can be found in Table 2 and Table 3.

**Figure 3.**
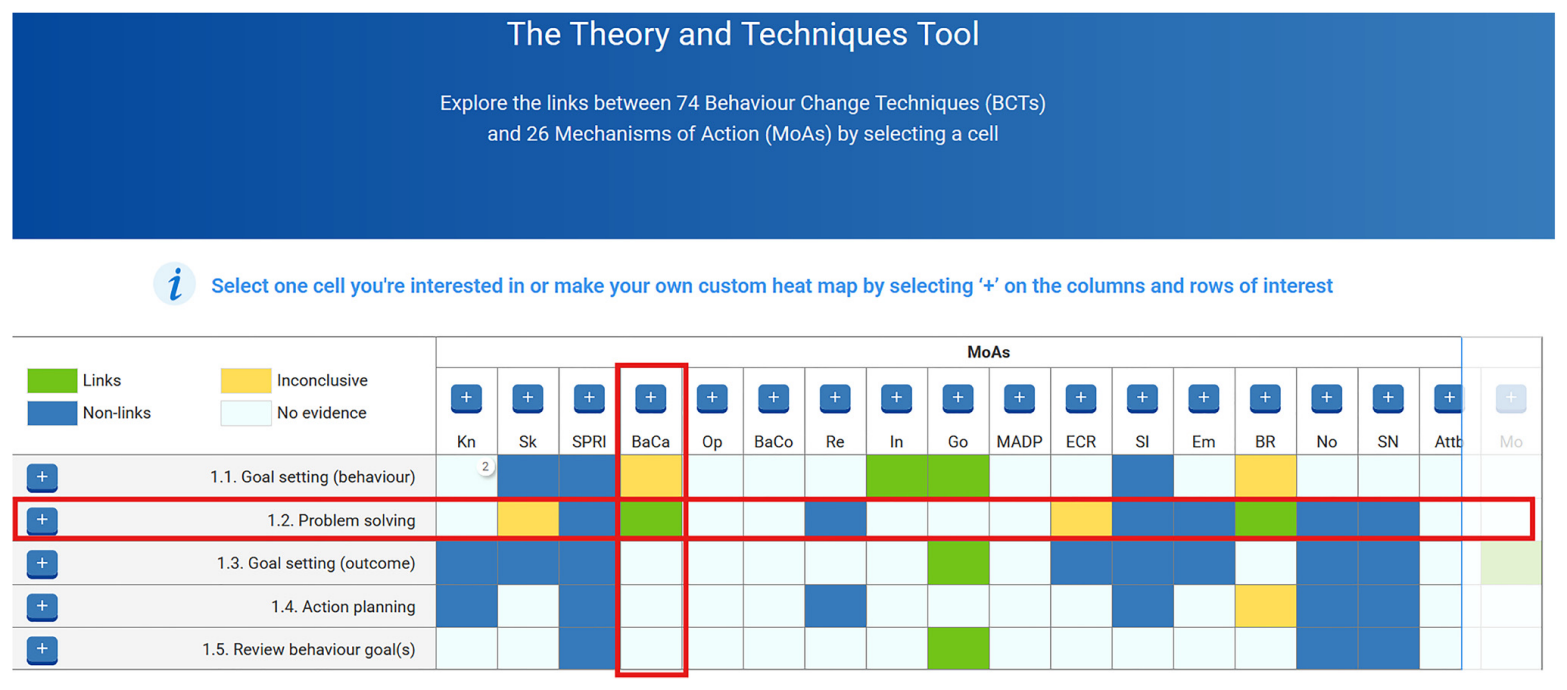
Screenshot of the Theory and Technique Tool (TaTT), with a BCT and MoA link highlighted.

To generate the BCT-MoA links in the TaTT, three studies were conducted ( Carey *et al*., 2019; Connell *et al*., 2019; Johnston *et al*., 2021). The first study was a literature synthesis that identified links between BCTs and MoAs in published intervention reports ( Carey *et al*., 2019), while the second was an expert consensus study where behaviour change experts rated BCT-MoA links ( Connell *et al*., 2019). A link was made when intervention reports included descriptions explicitly hypothesising that the BCT changes behaviour through the MoA, or behaviour change experts agreed that the BCT changed behaviour via the MoA. To triangulate these findings, a third study examined the concordance of links and reconciled discrepancies between these two sources of evidence ( Johnston *et al*., 2021). This triangulation proposed an evidence-based grid (a heat map) presenting links between 74 BCTs and 26 MoAs, which was made available in an online interactive platform ( https://theoryandtechniquetool.humanbehaviourchange.org/). The heat map contains 1924 cells (for every possible BCT-MoA link variation), with each cell colour coded indicating either a link (green), nonlink (blue), inconclusive (yellow), or lack of evidence (white) (see Figure 3). For example, in Figure 3, the red boxes signpost the BCT “1.2 Problem Solving” and the MoA “BaCa”, which stands for the “Belief about Capabilities”, while the green box in the grid indicates a link between this BCT and MoA. Clicking on any cell reveals the evidence for the relevant link from the three studies.

The TaTT can be used for several purposes, notably to:

- Identify evidence-based ‘likely’ BCT-MoA links to inform intervention development and evaluations
- Allow users to link BCTs in interventions, selected without an explicit basis in behaviour change theory, to the MoAs they likely target, facilitating these BCT-MoA links to be investigated and tested in future studies
- Maximise the rate of scientific advance by sharing data and knowledge as easily and efficiently as possible, by allowing users to submit new evidence to the tool about links.

### The Behaviour Change Technique Ontology and Mechanism of Action Ontology

Since the first release of the TaTT in 2018, there have been advances in structures for conceptualising and specifying behaviour change interventions, through the development of ***ontologies*** ( National Academies of Sciences, 2022). Ontologies are formal structures that represent knowledge within a domain in terms of uniquely specified ***classes*** of ***entities*** and ***relationships*** between them ( Arp *et al*., 2015; Hastings, 2017). An important feature of ontologies is that every class and type of relation between classes is given a unique ID in the form of a ***Uniform Resource Identifier*** (commonly referred to as ***URI***). This computational structure allows ontologies to be “read” by computers ( Arp *et al*., 2015; He *et al*., 2018; Seppälä *et al*., 2014); we can then use artificial intelligence approaches for automated processing of information, such as for evidence synthesis or predicting outcomes ( Hastings *et al*., 2023; West *et al*., 2024). Ontologies offer important benefits to advancing science. They facilitate:

- the accumulation of knowledge through interoperability (linking classes across domains and datasets) ( Baird *et al*., 2023; Hastings *et al*., 2024)
- more efficient information retrieval, data integration and data sharing ( Chen *et al*., 2010; Hastings *et al*., 2011)
- communication and collaboration across domains ( Gene Ontology Consortium, 2015; Sharp *et al*., 2023)

**Figure 4.**
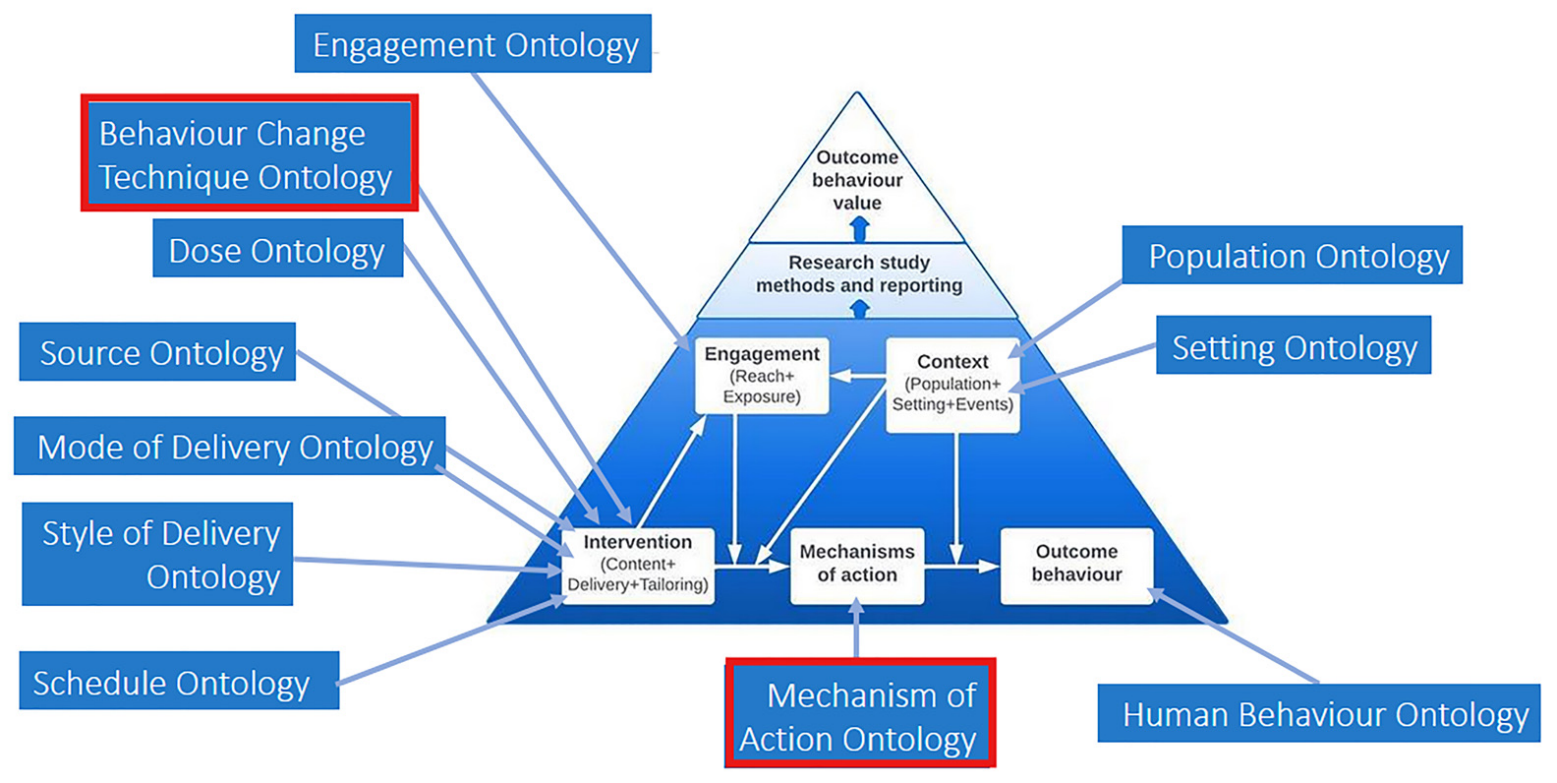
Schematic of the upper-level BCIO and its lower-level ontologies, with the red boxes around the Behaviour Change Technique Ontology (BCTO) and the Mechanism of Action (MoA) Ontology.

The development and use of ontologies in the behavioural and social sciences is growing ( Norris *et al*., 2019; Sharp *et al*., 2023). Most notably, the Behaviour Change Intervention Ontology (BCIO) has been recognised as an example of a detailed and precise ontology that is characterised by strong semantics ( National Academies of Sciences, 2022). The BCIO characterises behaviour change interventions, their MoAs, outcome behaviours, as well as engagement with interventions and intervention contexts, and the evaluations of interventions (see Figure 4; Michie *et al*., 2020). Figure 4 is a simplified schematic representation of the BCIO’s upper level, with upper-level classes shown in the white boxes. Each of these upper-level classes capture one or more ***lower-level ontologies*** part of the BCIO; these lower-level ontologies are signposted in the blue boxes, with the arrows indicating which broad class they relate to. For example, the box for “Intervention” captures an ontology for BCTs (called the ***Behaviour Change Techniques Ontology [BCTO])*** ( Marques *et al*., 2024b), as well as other ontologies for delivery, while the box for “Mechanism of action” captures the ***Mechanism of Action (MoA) Ontology*** ( Schenk *et al*., 2024b).

The links in the TaTT links relate to two classes within the upper-level BCIO and their relevant ontologies: BCTO and MoA Ontology (as shown in Figure 4). BCTO extends the BCTTv1 into a formal ontology ( Corker *et al*., 2023; Marques *et al*., 2024b), including most recently 285 BCTs. The Mechanism of Action Ontology ( Schenk *et al*., 2024b) specifies the potential processes of change in behaviour change interventions (potential MoAs) and includes 606 classes (last reported as 284 classes in Schenk *et al*. [2024b]), following an update informed by a recent mapping exercise of the BCIO to behavioural theories. Ontologies will continue to evolve in response to new evidence and feedback ( He *et al*., 2018), and the number of classes may increase in the future. The most up-to-date version of these ontologies can always be found and downloaded from the Human Behaviour-Change Project repository on ***GitHub***: https://github.com/HumanBehaviourChangeProject/ontologies

### Why align the TaTT with the BCTO and MoA Ontologies?

By using the TaTT alongside the BCTO and MoA Ontologies, researchers and practitioners could hypothesise potential links between the extended number of BCTs and MoAs in these ontologies. For example, starting with the ontology class for “self-efficacy belief for a behaviour” (alphanumeric ID: BCIO:006154), ontology users could explore potential links to BCTs through the TaTT, in this case looking at the links of the TaTT MoA “Belief about Capabilities”. A mapping between these tools can provide explicit guidance about how these tools could be used together and integrated.

Researchers and practitioners, who use the TaTT, could use a mapping to the ontologies to identify and report the more detailed, clearly defined ontology classes in their protocols and papers. In addition, the unique alphanumeric identifier (e.g. BCIO:006154) attached to each class allows data to be computer-readable, and thus enables further computational analysis ( Hastings, 2017; Matentzoglu *et al*., 2022). For example, by starting off with a TaTT MoA (e.g., “Memory, attention and decision process”) and then identifying the corresponding detailed ontology classes (e.g., “memory process” and “attending”), TaTT users can report more nuanced and varied evidence about BCT-MoA links or lack thereof. While ontologies facilitate being explicit and transparent about conceptual definitions, the BCTO and MoA Ontology are much more complex and time consuming to engage with than the TaTT. For TaTT users, a mapping to the ontologies can help them familiarise themselves with these new tools, without needing to immediately engage with the detailed ontologies.

Finally, an explicit mapping between the TaTT and ontologies will help users link and integrate evidence from studies using these two frameworks, thereby potentially feeding into a shared evidence base about behaviour change. In the future, this alignment could enable evidence accumulated with the TaTT to be used in machine learning applications, drawing on the computer-readable classes of the BCTO and MoA Ontology.

### Aim

This study aimed to create a mapping of the TaTT and the BCIO, in order for these tools to become more aligned for use in intervention development and evaluation. To achieve this, we mapped (1) the classes from the BCTO to one or more corresponding BCTs in the TaTT, and (2) the classes from the MoA Ontology to their corresponding MoAs in the TaTT.

## Methods

**Figure 5.**
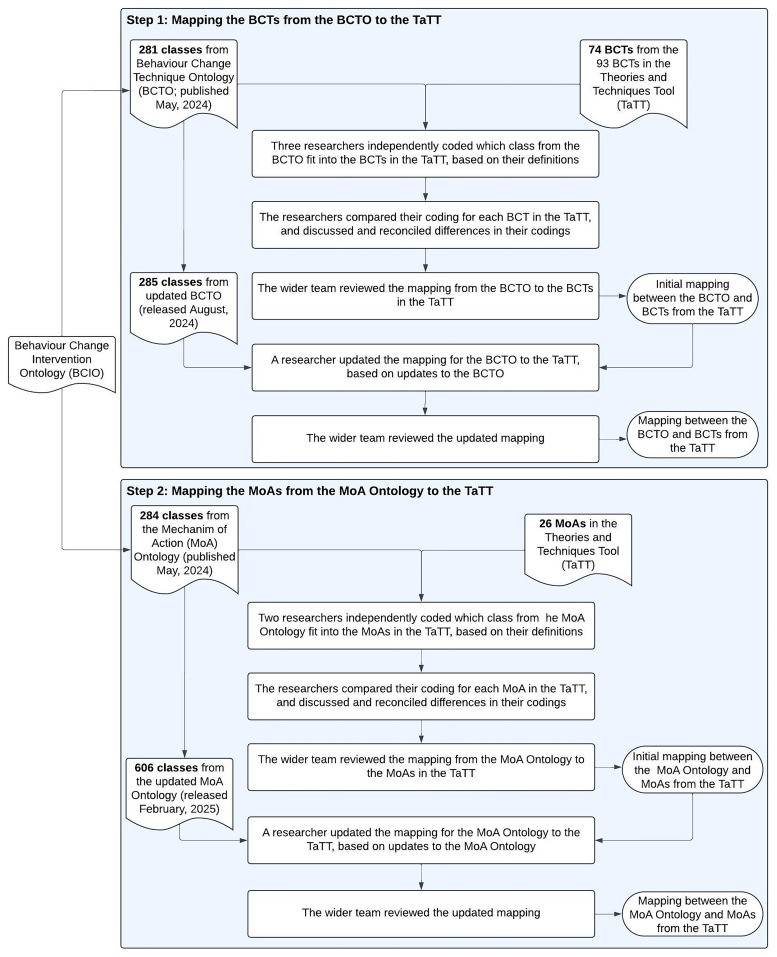
Overview of the steps to map the BCTO and MoA Ontology to the TaTT.

This study involved two steps: (1) mapping the BCTs (classes) from the BCTO ( Marques *et al*., 2024b) to the 74 BCTs in the TaTT ( Johnston *et al*., 2021) and (2) mapping MoAs (classes) from the MoA Ontology ( Schenk *et al*., 2024b) to the 26 MoAs in the TaTT ( Johnston *et al*., 2021). Figure 5 shows an overview of this process.

### Step 1: Mapping the BCTs from the BCTO to the TaTT

Three researchers (AW, MM, LZ) independently reviewed the 281 class labels and definitions in BCTO (published in May, 2024; see this version in https://osf.io/ya74q), judging and recording which classes were represented by each of the 74 BCTs in the TaTT ( https://theoryandtechniquetool.humanbehaviourchange.org/tool). For a class to be considered captured by a TaTT BCT, it needed to either (1) have a definition with the same meaning (a one-to-one match) or (2) include all the attributes of the BCT while providing more specific detail. In cases where a class did not align with a single TaTT BCT, the researchers recorded multiple TaTT BCTs for the class, as needed. They then compared their records, discussed any disagreements, and reconciled differences to finalise the mappings for the BCTs. The wider research team then reviewed these results and discussed whether additional classes from the ontology or new classes were needed to clearly capture any of the 74 BCTs from the TaTT.

Following this initial mapping, updates were made to the BCTO as part of another study (Michie *et al*., prep), resulting in four new classes being added. A researcher (LZ) updated the mapping to reflect the changes to the ontology (released August, 2024), recording relevant new classes for BCTs in the TaTT, and then verified the updated mapping with research group. The most recent version of the BCTO can be downloaded from https://github.com/HumanBehaviourChangeProject/ontologies/tree/master/BehaviourChangeTechniques.

### Step 2: Mapping the MoAs from the MoA Ontology to the TaTT

Two researchers (PS, MS) independently reviewed the 284 class labels and definitions in the MoA Ontology (published May, 2024; see this version in https://osf.io/pkq4e) and recorded which classes were captured by each of the 26 MoA groups in the TaTT ( https://theoryandtechniquetool.humanbehaviourchange.org/tool). For a class to be considered as captured by an MoA in the TaTT, the class definition needed to: (1) have an identical meaning to the TaTT MoA definition or (2) include all the attributes of the MoA’s definition while providing more specific detail. Unlike the BCT mapping, the researchers did not record multiple different TaTT MoAs for a single class from the MoA Ontology. We avoided this, as the MoA Ontology’s hierarchy and classes are complex, and a single class can include combinations of the MoAs in the TaTT. For example, the broad structural class “mental disposition” could include various different MoAs from the TaTT (e.g., “Knowledge”, “Attitudes”, “Belief about capability”). Therefore, to be more useable, this mapping was kept simpler.

After their independent coding, the researchers compared their coding, discussing and reconciling their disagreements to finalise their mapping. The wider research team then reviewed these results and discussed whether additional classes from the ontology or new classes were needed to clearly capture any groups.

The MoA mapping needed to be revised to reflect substantial changes to the MoA Ontology (released February, 2025 since its initial publication). A researcher (PS) reviewed the 322 new classes added to the ontology (with 606 classes in total) and recorded the relevant ones for MoA groups from the TaTT. The new additions were reviewed by the wider research group and added to the mapping based on their feedback. The most recent version of the BCTO can be downloaded from https://github.com/HumanBehaviourChangeProject/ontologies/tree/master/MechanismOfAction.

## Results

### Step 1: Mapping the BCTs from the BCTO to the TaTT

From the BCTO, 85 BCTs (classes) were, altogether, mapped onto the 74 BCTs from the TaTT. Of the BCTs in the BCTO, 59 had a one-to-one mapping to the BCTs listed in the TaTT. For example, the class “Goal strategising BCT [BCIO:007008]” corresponded to the “1.2 Problem solving” in the TaTT. As the BCTO contains more detailed BCTs compared to both BCTTv1 and the TaTT, multiple BCTO classes were mapped to 11 BCTs in the TaTT: nine TaTT BCTs each corresponded to two BCTO classes, while two TaTT BCTs corresponded to three BCTO classes. For example, the classes “Prompt intended action BCT [BCIO:007080]” and “Cue BCT [BCIO:007081]” were both mapped to “7.1 Prompts/cues” in the TaTT. Another key change in the BCTO from the BCTTv1 was no longer distinguishing between self- and other-enacted BCTs, as the source of an intervention is now specified through the Source Ontology ( Norris *et al*., 2021). This meant that the ontological class “Provide positive consequence for behaviour BCT” [BCIO:007252 (URI, i.e. alphanumeric ID)] was mapped onto the TaTT BCTs “10.3 Non-specific reward” and “10.9 Self-reward”. Similarly, the class “Promise positive consequence for behaviour BCT” [BCIO:007202] was mapped onto “10.6 Non-specific incentive” and “10.7 Self-incentive”. Table 2 presents the mapping. For reference, the earlier mapping of the BCTO (released May, 2024) can be found here: https://osf.io/r7cux)

**Table 2.** Mapping the 74 BCTs in the TaTT to the BCTs in the BCTO ( Johnston *et al*., 2018; Johnston *et al*., 2021; Marques *et al*., 2024b; Michie *et al*., 2013).

No.	BCT in the TaTT	Corresponding BCT classes in the BCT Ontology
1	**1.1 Goal setting (behaviour):** Set or agree on a goal defined in terms of the behaviour to be achieved	Set behaviour goal BCT [BCIO:007003] *: A <goal setting BCT> that sets a goal for the behaviour to be achieved. • **Set measurable behaviour goal BCT [BCIO:007300]:** A <set behaviour goal BCT> that describes the behaviour to be achieved in terms of a measurable target. **Agree behaviour goal BCT [BCIO:007004]**: A <goal setting BCT> that involves the intervention source agreeing with the person on a behavioural goal.
2	**1.2 Problem solving:** Analyse, or prompt the person to analyse, factors influencing the behaviour and generate or select strategies that include overcoming barriers and/or increasing facilitators	**Goal strategising BCT [BCIO:007008]:** A <goal directed BCT> in which the person analyses factors influencing the behaviour and generates, selects, or reviews strategies to increase facilitators and overcome barriers.
3	**1.3 Goal setting (outcome):** Set or agree on a goal defined in terms of a positive outcome of wanted behaviour	Set outcome goal BCT [BCIO:007005] *: A <goal setting BCT> in which the goal is a positive outcome of performing the behaviour. • **Set measurable outcome goal BCT [BCIO:007301]:** A <set outcome goal BCT> that describes the behavioural outcome to be achieved in terms of a measurable target. **Agree outcome goal BCT [BCIO:007006]**: A <goal setting BCT> that involves the intervention source agreeing with the person on a goal which is a positive outcome of performing the behaviour.
4	**1.4 Action planning:** Prompt detailed planning of performance of the behaviour (must include at least one of context, frequency, duration and intensity). Context may be environmental (physical or social) or internal (physical, emotional or cognitive)	**Action planning BCT [BCIO:007010]:** A <goal directed BCT> that involves making a detailed plan for the performance of the behaviour, which must include at least one of context, frequency, duration or intensity.
5	**1.5 Review behaviour goal(s):** Review behaviour goal(s) jointly with the person and consider modifying goal(s) or behaviour change strategy in light of achievement. This may lead to re-setting the same goal, a small change in that goal or setting a new goal instead of (or in addition to) the first, or no change.	**Review behaviour goal** **BCT [BCIO:007011]**: A <goal directed BCT> that reviews a behavioural goal and considers modifying the goal in light of progress toward the goal.
6	**1.6 Discrepancy between current behaviour and goal**: Draw attention to discrepancies between a person’s current behaviour (in terms of the form, frequency, duration, or intensity of that behaviour) and the person’s previously set outcome goals, behavioural goals or action plans (goes beyond self-monitoring of behaviour)	**Attend to discrepancy between current behaviour and goal BCT [BCIO:007012]:** A <goal directed BCT> that draws attention to discrepancies between a person's current behaviour and the person's outcome goal, behavioural goal or action plan.
7	**1.7 Review outcome goal(s):** Review outcome goal(s) jointly with the person and consider modifying goal(s) in light of achievement. This may lead to re-setting the same goal, a small change in that goal or setting a new goal instead of, or in addition to the first	**Review outcome goal** **BCT [BCIO:007013]**: A <goal directed BCT> that reviews an outcome goal and considers modifying the goal in light of achievement.
8	**1.8 Behavioural contract**: Create a written specification of the behaviour to be performed, agreed on by the person, and witnessed by another	**Create behavioural contract** **BCT [BCIO:007014]**: A <goal directed BCT> that creates a written specification of the behaviour to be performed, agreed on by the person, and witnessed by another person.
9	**1.9 Commitment**: Ask the person to affirm or reaffirm statements indicating commitment to change the behaviour	**Affirm commitment BCT [BCIO:007015]:** A <goal directed BCT> that asks the person to affirm or reaffirm statements indicating commitment to change the behaviour.
10	**2.1 Monitoring of behaviour by others without feedback**: Observe or record behaviour with the person’s knowledge as part of a behaviour change strategy	**Observe behaviour without feedback BCT [BCIO:007018]:** A <monitoring BCT> that monitors current performance of the behaviour with the person’s knowledge but without providing feedback about their behaviour. **Record behaviour without feedback BCT [BCIO:007019]:** A <monitoring BCT> that documents current performance of the behaviour with the person’s knowledge but without providing feedback about their behaviour.
11	**2.2 Feedback on behaviour:** Monitor and provide informative or evaluative feedback on performance of the behaviour *(e.g. form, frequency, duration, intensity)*	**Provide feedback on behaviour** **BCT [BCIO:007023]**: A <provide feedback BCT> that provides information about the person's previous performance of the behaviour.
12	**2.3 Self-monitoring of behaviour:** Establish a method for the person to monitor and record their behaviour(s) as part of a behaviour change strategy	**Self-monitor behaviour** **BCT [BCIO:007024]**: A <monitoring BCT> in which the person uses a method to monitor and record their behaviour.
13	**2.4 Self-monitoring outcome(s) of behaviour:** Establish a method for the person to monitor and record the outcome(s) of their behaviour as part of a behaviour change strategy	**Self-monitor outcome of behaviour** **BCT [BCIO:007025]**: A <monitoring BCT> in which the person uses a method to monitor and record an outcome of their behaviour.
14	**2.5 Monitoring of outcome(s) of behaviour by others without feedback:** Observe or record outcomes of behaviour with the person’s knowledge as part of a behaviour change strategy	**Observe outcome of behaviour without feedback BCT [BCIO:007020]:** A <monitoring BCT> that monitors an outcome of performing the behaviour with the person’s knowledge but without providing feedback about the outcome. **Record outcome of behaviour without feedback BCT [BCIO:007021]:** A <monitoring BCT> that documents an outcome of performing the behaviour with the person's knowledge but without providing feedback about the outcome.
15	**2.6 Biofeedback:** Provide feedback about the body *(e.g. physiological or biochemical state)* using an external monitoring device as part of a behaviour change strategy	**Provide biofeedback BCT [BCIO:007026]:** A <provide feedback BCT> that provides information about the functioning or state of the person's body, based on information collected by an external monitoring device.
16	**2.7 Feedback on outcome(s) of behaviour:** Monitor and provide feedback on the outcome of performance of the behaviour	**Provide feedback on outcome of behaviour BCT [BCIO:007027]:** A <provide feedback BCT> that provides information about an outcome of the person's previous performance of the behaviour.
17	**3.1 Social support (unspecified):** Advise on, arrange or provide social support *(e.g. from friends, relatives, colleagues,’ buddies’ or staff)* or non-contingent praise or reward for performance of the behaviour *.* It includes encouragement and counselling, but only when it is directed at the behaviour	Social support BCT [BCIO:007028] *: A <behaviour change technique> that involves taking steps to secure or deliver the support or aid of another person. • **Advise to seek support BCT [BCIO:007029]:** A <social support BCT> that involves advising the person to seek support from another person. • **Arrange support BCT [BCIO:007034]:** A <social support BCT> that organises support from another for the person. • **Deliver support BCT [BCIO:007039]:** A <social support BCT> that directly provides support to the person.
18	**3.2 Social support (practical):** Advise on, arrange, or provide practical help *(e.g. from friends, relatives, colleagues, ‘buddies’ or staff)* for performance of the behaviour	**Advise to seek instrumental support BCT [BCIO:007030]:** An <advise to seek support BCT> that suggests the person try to obtain support from another in terms of tangible aid. **Arrange instrumental support BCT [BCIO:007035]:** An <arrange support BCT> that organises support from another in terms of tangible aid. **Deliver instrumental support BCT [BCIO:007040]:** A <deliver support BCT> that provides tangible aid.
19	**3.3 Social support (emotional):** Advise on, arrange, or provide emotional social support *(e.g. from friends, relatives, colleagues, ‘buddies’ or staff)* for performance of the behaviour	**Advise to seek emotional support BCT [BCIO:007031]:** An <advise to seek support BCT> that suggests the person try to obtain support from another in terms of expressing concern, caring and empathy. **Arrange emotional support BCT [BCIO:007036]:** An <arrange support BCT> that organises support from another in terms of expressing concern, caring and empathy. **Deliver emotional support BCT [BCIO:007041]:** A <deliver support BCT> that provides expressions of concern, caring and empathy.
20	**4.1 Instruction on how to perform behaviour:** Advise or agree on how to perform the behaviour	**Instruct how to perform behaviour BCT [BCIO:007058]:** A <guide how to perform behaviour BCT> that involves telling the person how to perform the behaviour. **Agree on how to perform behaviour BCT [BCIO:007051]:** A <guide how to perform behaviour BCT> that involves reaching consensus on how to perform the behaviour.
21	**4.2 Information about antecedents:** Provide information about antecedents ( *e.g. social and environmental situations and events, emotions, cognitions)* that reliably predict performance of the behaviour	**Inform about antecedents BCT [BCIO:007052]:** A <suggest different perspective on behaviour BCT> that involves providing factual information to the person regarding triggers or influences that precede the initiation of the behaviour.
22	**4.3 Re-attribution:** Elicit perceived causes of behaviour and suggest alternative explanations *(e.g. external or internal and stable or unstable)*	**Re-attribute cause BCT [BCIO:007053]:** A <suggest different perspective on behaviour BCT> that involves eliciting the person's beliefs about, and suggesting alternative beliefs about, the causes of the behaviour.
23	**5.1 Information about health consequences:** Provide information (e.g. written, verbal, visual) about health consequences of performing the behaviour	Inform about health consequences BCT [BCIO:007063] *: An <increase awareness of consequences BCT> that provides information about the physical or mental health consequences of performing or not performing the behaviour. • **Inform about positive health consequences BCT [BCIO:007183]:** An <inform about health consequences BCT> that provides information about the positive physical or mental health consequences of performing or not performing the behaviour. • **Inform about negative health consequences BCT [BCIO:007179]**: An <inform about health consequences BCT> that provides information about the negative physical or mental health consequences of performing or not performing the behaviour.
24	**5.2 Salience of consequences:** Use methods specifically designed to emphasise the consequences of performing the behaviour with the aim of making them more memorable (goes beyond informing about consequences)	**Increase salience of consequences BCT [BCIO:007068]:** An <increase awareness of consequences BCT> that emphasises the consequences in a way that makes them more vivid or emotionally-laden.
25	**5.3 Information about social and environmental consequences:** Provide information (e.g. written, verbal, visual) about social and environmental consequences of performing the behaviour	Inform about social consequences BCT [BCIO:007064] *: An <increase awareness of consequences BCT> that provides information about the social consequences of performing or not performing the behaviour. • **Inform about positive social consequences BCT [BCIO:007184]**: An <inform about social consequences BCT> that provides information about the positive social consequences of performing or not performing the behaviour. • **Inform about negative social consequences BCT [BCIO:007180]:** An <inform about social consequences BCT> that provides information about the negative social consequences of performing or not performing the behaviour. Inform about environmental consequences BCT [BCIO:007176] *: An <increase awareness of consequences BCT> that provides information about the environmental consequences of performing or not performing the behaviour. • **Inform about positive environmental consequences BCT [BCIO:007182]:** An <inform about environmental consequences BCT> that provides information about the positive environmental consequences of performing or not performing the behaviour. • **Inform about negative environmental consequences BCT [BCIO:007178]:** An <inform about environmental consequences BCT> that provides information about the negative environmental consequences of performing or not performing the behaviour.
26	**5.4 Monitoring of emotional consequences**: Prompt assessment of feelings after attempts at performing the behaviour	**Monitor emotional consequences BCT [BCIO:007066]:** A <monitoring BCT> that involves the person assessing their emotions after performing the behaviour
27	**5.5 Anticipated regret:** Induce or raise awareness of expectations of future regret about performance of the unwanted behaviour	**Induce anticipated regret BCT [BCIO:007067]:** An <inform about emotional consequences BCT> that focuses on expectations of remorse after performing or not performing the behaviour.
28	**5.6 Information about emotional consequences:** Provide information (e.g. written, verbal, visual) about emotional consequences of performing the behaviour	Inform about emotional consequences BCT [BCIO:007065] *: An <increase awareness of consequences BCT> that provides information about the emotional consequences of performing or not performing the behaviour. • **Inform about positive emotional consequences BCT [BCIO:007181]:** An <inform about emotional consequences BCT> that provides information about the positive emotional consequences of performing or not performing the behaviour. • **Inform about negative emotional consequences BCT [BCIO:007177]:** An <inform about emotional consequences BCT> that provides information about the negative emotional consequences of performing or not performing the behaviour.
29	**6.1 Demonstration of the behaviour:** Provide an observable sample of the performance of the behaviour, directly in person or indirectly e.g. via film, pictures, for the person to aspire to or imitate	**Demonstrate the behaviour BCT [BCIO:007055]:** A <guide how to perform behaviour BCT> that provides an observable sample of the performance of the behaviour for the person to aspire to or imitate.
30	**6.2 Social comparison:** Draw attention to others’ performance to allow comparison with the person’s own performance	**Prompt social comparison BCT [BCIO:007073]:** An <awareness of other people’s thoughts, feelings and actions BCT> that draws attention to other people’s behaviour and compares it with the person’s own behaviour.
31	**6.3 Information about others’ approval:** Provide information about what other people think about the behaviour. The information clarifies whether others will like, approve or disapprove of what the person is doing or will do	**Increase awareness of others’ approval BCT [BCIO:007074]:** An <awareness of other people’s thoughts, feelings and actions BCT> that increases awareness of whether others will like, approve, dislike, or disapprove of the behaviour.
32	**7.1 Prompts/cues:** Introduce or define environmental or social stimulus with the purpose of prompting or cueing the behaviour. The prompt or cue would normally occur at the time or place of performance	**Prompt intended action BCT [BCIO:007080]:** An <alter external stimulus BCT> that involves introducing an external stimulus to facilitate the behaviour for which an intention has previously been formed. **Cue BCT [BCIO:007081]:** An <alter external stimulus BCT> that introduces external information that is already associated with the behaviour in order to elicit that behaviour.
33	**7.5 Remove aversive stimulus:** Advise or arrange for the removal of an aversive stimulus to facilitate behaviour change	**Remove aversive stimulus BCT [BCIO:050331]:** An <alter external stimulus BCT> that involves removing an aversive stimulus to bring about behaviour change.
34	**7.7 Exposure:** Provide systematic confrontation with a feared stimulus to reduce the response to a later encounter	**Expose to sustained aversive stimulus BCT [BCIO:007170]:** An <expose to stimulus BCT> that involves sustained exposure to an aversive stimulus to reduce the likelihood of the behaviour when encountering that stimulus. **Gradually increase exposure to aversive stimulus BCT [BCIO:007172]**: An <expose to stimulus BCT> that involves gradually increasing exposure to an aversive stimulus to reduce the likelihood of the behaviour when encountering that stimulus.
35	**7.8 Associative learning:** Present a neutral stimulus jointly with a stimulus that already elicits the behaviour repeatedly until the neutral stimulus elicits that behaviour	Associative learning BCT [BCIO:007090] **: A <behaviour change technique> that involves repeated pairing of a stimulus with another stimulus or with a behavioural outcome.
36	**8.1 Behavioural practice/rehearsal:** Prompt practice or rehearsal of the performance of the behaviour one or more times in a context or at a time when the performance may not be necessary, in order to increase habit and skill	**Practise behaviour BCT [BCIO:007094]:** An <advise specific behaviour BCT> that advises repetition of the behaviour in a way that has the function of increasing the skill in performing the behaviour.
37	**8.2 Behaviour substitution:** Prompt substitution of the unwanted behaviour with a wanted or neutral behaviour	**Substitute behaviour BCT [BCIO:007095]:** An <advise specific behaviour BCT> that advises the person to replace the unwanted behaviour with another behaviour.
38	**8.3 Habit formation:** Prompt rehearsal and repetition of the behaviour in the same context repeatedly so that the context elicits the behaviour	**Context-specific repetition of behaviour BCT [BCIO:007096]:** An <advise specific behaviour BCT> that advises the person to repeat the behaviour in the same context.
39	**8.4 Habit reversal:** Prompt rehearsal and repetition of an alternative behaviour to replace an unwanted habitual behaviour	**Context-specific repetition of alternative behaviour BCT [BCIO:007097]:** An <advise specific behaviour BCT> that advises the person to repeat an alternative behaviour consistently in a context that previously elicited an unwanted behaviour.
40	**8.6 Generalisation of target behaviour:** Advise to perform the wanted behaviour, which is already performed in a particular situation, in another situation	**Generalise behaviour BCT [BCIO:007099]:** An <advise specific behaviour BCT> that advises the person to perform the behaviour which is already performed in a particular context, in a similar context.
41	**8.7 Graded tasks:** Set easy-to-perform tasks, making them increasingly difficult, but achievable, until behaviour is performed	**Set graded tasks BCT [BCIO:007100]:** A <goal directed BCT> that sets easy-to-perform tasks for the person, making them increasingly difficult, but achievable, until the behaviour is performed.
42	**9.1 Credible source:** Present verbal or visual communication from a credible source in favour of or against the behaviour	**Present information from credible influence BCT [BCIO:007075]:** An <awareness of other people’s thoughts, feelings and actions BCT> that presents information from a credible person or organisation to influence the behaviour.
43	**9.2 Pros and cons:** Advise the person to identify and compare reasons for wanting (pros) and not wanting to (cons) change the behaviour (includes ‘ **Decisional balance’** )	**Consider pros and cons BCT [BCIO:007069]:** An <increase awareness of consequences BCT> that advises identification and comparison of the positive and negative consequences of performing or not performing the behaviour.
44	**9.3 Comparative imagining of future outcomes:** Prompt or advise the imagining and comparing of future outcomes of changed versus unchanged behaviour	**Prompt comparative imagining of future outcomes BCT [BCIO:007070]:** An <increase awareness of consequences BCT> that guides the person to imagine and compare the consequences of performing and not performing the behaviour.
45	**10.1 Material incentive (behaviour):** Inform that money, vouchers or other valued objects *will be* delivered if and only if there has been effort and/or progress in performing the behaviour (includes *‘* **Positive reinforcement’**)	**Promise positive material consequence for behaviour BCT [BCIO:007209]:** A <promise positive consequence for behaviour BCT> where the consequence is money, vouchers or other valued objects.
46	**10.2 Material reward (behaviour):** Arrange for the delivery of money, vouchers or other valued objects if and only if there *has been* effort and/or progress in performing the behaviour (includes ‘ **Positive reinforcement’**)	**Provide positive material consequence for behaviour BCT [BCIO:007257]:** A <provide positive consequence for behaviour BCT> where the consequence is money, vouchers or other valued objects.
47	**10.3 Non-specific reward:** Arrange delivery of a reward if and only if there *has been* effort and/or progress in performing the behaviour (includes ‘ **Positive reinforcement’**)	Provide positive consequence for behaviour BCT [BCIO:007252] **: A <provide consequence for behaviour BCT> where the consequence is positive.
48	**10.4 Social reward:** Arrange verbal or non-verbal reward if and only if there *has been* effort and/or progress in performing the behaviour (includes ‘ **Positive reinforcement**’)	**Provide positive social consequence for behaviour BCT [BCIO:007265]:** A <provide positive consequence for behaviour BCT> where the consequence is an interpersonal process or a proxy interpersonal process.
49	**10.6 Non-specific incentive:** Inform that a reward *will be* delivered if and only if there has been effort and/or progress in performing the behaviour (includes ‘ **Positive reinforcement’**)	Promise positive consequence for behaviour BCT [BCIO:007202] **: A <promise consequence for behaviour BCT> where the consequence is positive.
50	**10.7 Self-incentive:** Plan to reward self in future if and only if there has been effort and/or progress in performing the behaviour	The BCTO no longer distinguishes between self- and other-enacted BCTs so this BCT is mapped to: Promise positive consequence for behaviour BCT [BCIO:007202] **: A <promise consequence for behaviour BCT> where the consequence is positive.
51	**10.8 Incentive (outcome):** Inform that a reward *will be* delivered if and only if there has been effort and/or progress in achieving the behavioural outcome ( *includes ‘* **Positive reinforcement** *’*)	Promise positive consequence for outcome of behaviour BCT [BCIO:007216] *: A <promise consequence for outcome of behaviour BCT> where the consequence is positive • **Promise positive social consequence for outcome of behaviour BCT [BCIO:007224]:** A <promise positive consequence for outcome of behaviour BCT> in which the consequence is an interpersonal process or a proxy interpersonal process. • **Promise positive material consequence for outcome of behaviour BCT [BCIO:007215]:** A <promise positive consequence for outcome of behaviour BCT> in which the consequence is money, vouchers or other valued objects.
52	**10.9 Self-reward:** Prompt self-praise or self-reward if and only if there *has been* effort and/or progress in performing the behaviour	The BCTO no longer distinguishes between self- and other-enacted BCTs so this BCT is mapped to: Provide positive consequence for behaviour BCT [BCIO:007252] **: A <provide consequence for behaviour BCT> where the consequence is positive.
53	**10.10 Reward (outcome):** Arrange for the delivery of a reward if and only if there *has been* effort and/or progress in achieving the behavioural outcome (includes ‘ **Positive reinforcement**’)	Provide positive consequence for outcome of behaviour BCT [BCIO:007264] **: A <provide consequence for outcome of behaviour BCT> where the consequence is positive.
54	**11.1 Pharmacological support:** Provide, or encourage the use of or adherence to, drugs to facilitate behaviour change	Promote pharmacological support BCT [BCIO:007144] *: A <behaviour change technique> promoting medicines or other drugs. • **Provide pharmacological support BCT [BCIO:007145]:** A <promote pharmacological support BCT> that provides the person with medicines or other drugs. • **Encourage pharmacological support BCT [BCIO:007146]:** A <promote pharmacological support BCT> that encourages the person to use medicines or other drugs.
55	**11.2 Reduce negative emotions:** Advise on ways of reducing negative emotions to facilitate performance of the behaviour (includes ‘ **Stress Management**’)	**Advise how to reduce negative emotions BCT [BCIO:050344]:** An <advise how to change emotions BCT> suggesting a method to decrease negative emotions.
56	**11.3 Conserving mental resources:** Advise on ways of minimising demands on mental resources to facilitate behaviour change	**Conserve mental resources BCT [BCIO:007134]:** A <manage mental processes BCT> that advises a way to minimise demands on mental resources.
57	**11.4 Paradoxical instructions:** Advise to engage in some form of the unwanted behaviour with the aim of reducing motivation to engage in that behaviour	**Advise paradoxical behaviour BCT [BCIO:007135]:** An <advise specific behaviour BCT> that advises the person to engage in an unwanted behaviour in a way that is aversive.
58	**12.1 Restructuring the physical environment:** Change, or advise to change the physical environment in order to facilitate performance of the wanted behaviour or create barriers to the unwanted behaviour (other than prompts/cues, rewards and punishments)	Restructure the physical environment BCT [BCIO:050348] **: A <restructure the environment BCT> that alters the physical environment in which the behaviour is, or would have been, performed in a way that facilitates or impedes the behaviour.
59	**12.2 Restructuring the social environment:** Change, or advise to change the social environment in order to facilitate performance of the wanted behaviour or create barriers to the unwanted behaviour (other than prompts/cues, rewards and punishments)	Restructure the social environment BCT [BCIO:050349] *: A <restructure the environment BCT> that alters the social environment in which the behaviour is, or would have been, performed in a way that facilitates or impedes the behaviour. • **Directly restructure the social environment BCT [BCIO:050346]**: A <restructure the social environment BCT> that changes the person’s directly experienced environment at the time the behaviour is, or would have been, performed. • **Indirectly restructure the social environment BCT [BCIO:050347]:** A <restructure the social environment BCT> that changes the person’s environment at a time or location other than when and where the behaviour is performed.
60	**12.3 Avoidance/reducing exposure to cues for the behaviour:** Advise on how to avoid exposure to specific social and contextual/physical cues for the behaviour, including changing daily or weekly routines	**Reduce exposure to cues for the behaviour BCT [BCIO:007153]:** An <alter external stimulus BCT> that reduces an external stimulus that signals the behaviour.
61	**12.5 Adding objects to the environment:** Add objects to the environment in order to facilitate performance of the behaviour	Add objects to the environment BCT [BCIO:007156] *: An <environmental restructuring BCT> that adds objects to the person’s physical surroundings. • **Add objects to the directly experienced environment BCT [BCIO:007163]:** An <add objects to the environment BCT> that adds an object to the person’s directly experienced environment at the time the behaviour is, or would have been, performed. • **Add objects to the indirectly experienced environment BCT [BCIO:007164]:** An <add objects to the environment BCT> that adds an object to the person’s environment at a time or location other than when and where the behaviour is performed.
62	**12.6 Body changes:** Alter body structure, functioning or support directly to facilitate behaviour change	**Change the body BCT [BCIO:007136]:** A <behaviour change technique> that alters the structure or functioning of the person’s body.
63	**13.1 Identification of self as role model:** Inform that one’s own behaviour may be an example to others	**Identify self as role model BCT [BCIO:007158]:** A <prompt focus on self-identity BCT> that informs the person that their behaviour may be an example to others.
64	**13.2 Framing/reframing:** Suggest the deliberate adoption of a perspective or new perspective on behaviour (e.g. its purpose) in order to change cognitions or emotions about performing the behaviour (includes ‘ **Cognitive structuring**’)	**Reframe past behaviour BCT [BCIO:007056]:** A <suggest different perspective on behaviour BCT> that involves reattributing a person’s successes to internal, stable or global factors or failures to external, unstable or specific factors.
65	**13.3 Incompatible beliefs:** Draw attention to discrepancies between current or past behaviour and self-image, in order to create discomfort (includes *‘* **Cognitive dissonance’**)	**Draw attention to incompatible beliefs BCT [BCIO:007057]:** A <suggest different perspective on behaviour BCT> that draws the person’s attention to the discrepancies between current or past behaviour and self-identity.
66	**13.4 Valued self-identity:** Advise the person to write or complete rating scales about a cherished value or personal strength as a means of affirming the person’s identity as part of a behaviour change strategy (includes *‘* **Self-affirmation’**)	**Affirm valued self-identity BCT [BCIO:007159]:** A <prompt focus on self-identity BCT> that advises engagement in activities that affirm the person’s valued attributes.
67	**13.5 Identity associated with changed behaviour:** Advise the person to construct a new self-identity as someone who ‘used to engage with the unwanted behaviour’	**Adopt changed self-identity BCT [BCIO:007160]:** A <prompt focus on self-identity BCT> that promotes the adoption of a self-identity as someone who engages in the behaviour that is different from their previous behaviour.
68	**14.2 Punishment:** Arrange for aversive consequence contingent on the performance of the unwanted behaviour	Provide aversive consequence for behaviour BCT [BCIO:007241] **: A <provide consequence for behaviour BCT> where the consequence is aversive.
69	**15.1 Verbal persuasion about capability:** Tell the person that they can successfully perform the wanted behaviour, arguing against self-doubts and asserting that they can and will succeed	**Persuade about personal capability BCT [BCIO:007137]:** A <prompt thinking related to successful performance BCT> that persuades the person that they can successfully perform the behaviour.
70	**15.2 Mental rehearsal of successful performance:** Advise to practise imagining performing the behaviour successfully in relevant contexts	**Prompt mental rehearsal of successful performance BCT [BCIO:007138]:** A <prompt thinking related to successful performance BCT> that prompts the person to practise imagining performing the behaviour well in a relevant context.
71	**15.3 Focus on past success:** Advise to think about or list previous successes in performing the behaviour (or parts of it)	**Prompt focus on past success BCT [BCIO:007139]:** A <prompt thinking related to successful performance BCT> that prompts the person to think about previous successful performance of the behaviour.
72	**15.4 Self-talk:** Prompt positive self-talk (aloud or silently) before and during the behaviour	**Prompt self-talk BCT [BCIO:007140]:** A <prompt thinking related to successful performance BCT> that promotes the use of positive self-talk before or during the behaviour.
73	**16.2 Imaginary reward:** Advise to imagine performing the **wanted** behaviour in a real-life situation followed by imagining a pleasant consequence (includes *‘* **Covert conditioning’**)	**Imagine reward BCT [BCIO:007119]:** An <increase awareness of consequences BCT> that guides the person to imagine performing the wanted behaviour in a real-life situation followed by experiencing a pleasant consequence for performing that behaviour.
74	**16.3 Vicarious consequences:** Prompt observation of the consequences (including rewards and punishments) for others when they perform the behaviour	**Vicarious reward BCT [BCIO:007120]:** An <increase awareness of consequences BCT> that prompts observation of another person being rewarded when they perform the behaviour. **Vicarious punishment BCT [BCIO:007121]:** An <increase awareness of consequences BCT> that prompts observation of another person being punished when they perform the behaviour.

*Note*. BCT = Behaviour Change Technique; BCTO = Behaviour Change Technique Ontology; TaTT = Theory and Technique Tool * In these cases, both a class and its subclasses are shown in the mapping. This was done as the subclasses were considered to capture important aspects of a BCT group and would be useful to view in the mapping. ** This BCT has a large number of child classes – please refer to the full BCTO ( https://github.com/HumanBehaviourChangeProject/ontologies/blob/master/BehaviourChangeTechniques/BCIO-bcto-hierarchy.xlsx) for details

### Step 2: Mapping the MoAs from the MoA Ontology to the TaTT

Drawing on the MoA Ontology’s most recent version (released February, 2025), 56 classes (not counting their subclasses) were, altogether, mapped onto the 26 MoAs (1-5 classes per MoA in the TaTT). Eight ontology classes had a one-to-one mapping to TaTT MoAs, such as the class “Knowledge” (BCIO:00605) class corresponding to the TaTT MoA “Knowledge”. Since the ontology included more specific classes than the MoAs in the TaTT, each of the remaining 18 MoAs in the TaTT corresponded to two to five classes. For example, the following ontology classes were mapped onto the broader TaTT MoA “Memory, attention & decision processes”: “Memory process” [BCIO:050319], “Attending” [MF:0000018], “Attentional disposition” [BCIO:050572] and “Decision-making” [BCIO:006116]. The complete mapping can be seen in Table 3.

**Table 3.** Mapping the 26 MoAs in the TaTT to the MoAs in the MoA Ontology ( Johnston *et al*., 2018; Johnston *et al*., 2021; Schenk *et al*., 2024b).

No.	MoA in the TaTT	Corresponding MoA classes in the MoA Ontology
1	**Knowledge**: An awareness of the existence of something	**Knowledge [BCIO:00605]**: A <mental disposition> to understand the nature of the world, or a specific aspect of the world, that corresponds to the actual state of the world and is acquired through experience or learning.
2	**Skill**: An ability or proficiency acquired through practice.	**Mental skill [BCIO:006004]**: A <mental capability> acquired through training or practice. **Self-regulatory skill [BCIO:050222]**: A <self-regulation capability> that is acquired through training or practice. **Physical skill** **[BCIO:006010]**: A <physical behavioural capability> acquired through training or practice. **Social skill [BCIO:006012]**: A <social behavioural capability> acquired through training or practice.
3	**Social/Professional role & identity**: A coherent set of behaviours and displayed personal qualities of an individual in a social or work setting	Personal role [BCIO:006081] *: A <role> that inheres in a human being by virtue of their social and institutional circumstances. • **Occupational role [BCIO:015430]:** A <personal role> that is realised in a person by doing a specified type of work or working in a specified way. • **Social role [BCIO:006082]:** A <personal role> that is realised in human social processes. **Identity** [ADDICTO:0000381] *: A <cognitive representation> of themselves by a person or group. • **Self-identity [ADDICTO:0000399]:** An <identity> that a person has about themselves. ◦ **Professional identity [BCIO:050229]:** A <self-identity> that is associated with one's occupational role. ◦ **Social identity [ADDICTO:0001087]:** A <self-identity> that represents a relation between oneself and another person or group • **Group identity [ADDICTO:0000715]:** An <identity> that a group holds about itself.
4	**Beliefs about capabilities:** Beliefs about one’s ability to successfully carry out a behaviour.	**Self-efficacy belief for a behaviour and its associated outcomes [BCIO:006043]:** A <self-efficacy belief> to organise and execute a behaviour and achieve the outcomes associated with this behaviour. **Self-efficacy belief for a behaviour [BCIO:006154]**: A <self-efficacy belief> to organise and execute a behaviour.
5	**Optimism:** Confidence that things will happen for the best or that desired goals will be attained.	**Belief about likelihood of consequences of an occurrence [BCIO:006026]:** A <belief> in terms the probability that a given event or state will occur or not occur in the future. **Evaluative belief [BCIO:006038]:** A <belief> about whether a particular aspect of the world is positive or negative.
6	**Beliefs about consequences:** Beliefs about the consequences of a behaviour (i.e. perceptions about what will be achieved and/or lost by undertaking a behaviour, as well as the probability that a behaviour will lead to a specific outcome).	**Belief about consequences of behaviour [BCIO:006019]:** A < belief about consequences of an occurrence > in terms of what results from or follows the performance of a behaviour. Consequences can be either positive or negative. **Belief about likelihood of consequences of behaviour [BCIO:006024]:** A <belief about likelihood of consequences of an occurrence> in terms of the probability that a behaviour will result or not result in particular outcomes.
7	**Reinforcement:** Processes by which the frequency or probability of a response is increased through a dependent relationship or contingency with a stimulus or circumstance.	**Internal reward for a response [BCIO:006100]:** A <bodily process> by which the person experiences an internally-generated positive physical or psychological state subsequent to a response. **Reinforcement process [BCIO:050755]:** A <process> in which a behaviour is followed by an event that alters the likelihood of occurrence of the behaviour.
8	**Intention:** A conscious decision to perform a behaviour or a resolve to act in a certain way.	**Behavioural intention [BCIO:006016]:** A <mental disposition> to commit to enact or not enact a behaviour.
9	**Goals:** Mental representations of outcomes or end states that an individual wants to achieve.	**Goal [BCIO:006049]:** A <cognitive representation> of an end state towards which one is striving.
10	**Memory, attention & decision processes:** Ability to retain information, focus on aspects of the environment and choose between two or more alternatives.	**Memory process [BCIO:050319]:** A <mental process> that is the encoding, storing, and retrieval of informational stimuli. **Attending [MF:0000018]:** A <mental process> whereby relevant aspects of one's mental experience are focused on specific targets. **Attentional disposition [BCIO:050572]:** A <mental disposition> that is realised by focusing one's attention on events, objects, sensory patterns or cognitive representations. **Decision-making [BCIO:006116]: **<Judging> in which one or more propositions or behaviours are identified as preferred from a larger number.
11	**Environmental context & resources:** Aspects of a person’s situation or environment that discourage or encourage the behaviour.	**Environmental system [ENVO:01000254]:** A <system> which has the disposition to surround and interact with one or more material entities. Environmental disposition [ENVO:01000452] *: A disposition which is realised by an environmental system or system parts thereof. • **Behavioural opportunity [BCIO:006086]:** An <environmental disposition> that is required for or facilitates a behaviour.
12	**Social influences:** Those interpersonal processes that can cause oneself to change one’s thoughts, feelings or behaviours	Socially-related behaviour [BCIO:050441] *: An <individual human behaviour> that relates to the social environment. • **Inter-personal behaviour [BCIO:036025]:** A < socially-related behaviour> that involves an interaction between two or more people. ◦ **Social influence behaviour [BCIO:006099]:** An <inter-personal behaviour> where a person exerts an influence on the behaviour of another. **Interpersonal process** [MF:0000021] *: A <bodily process> in which at least two human beings are agents. • **Social influence process [BCIO:050776]:** An <interpersonal process> in which people’s thoughts, feelings or behaviours are influenced by other people.
13	**Emotion:** A complex reaction pattern involving experiential, behavioural, and physiological elements.	**Emotion process [MFOEM:000001]:** An <affective process> that is a synchronized aggregate of constituent mental processes, including an appraisal process, which is valanced, has an object, and gives rise to an action tendency.
14	**Behavioural regulation:** Behavioural, cognitive and/or emotional skills for managing or changing behaviour.	**Self-regulation capability [BCIO:006005]:** A <mental capability> that involves processes that modulate the frequency, rate or extent of a response to external or internal stimuli and that are instigated by the person themselves. **Self-regulation of behaviour [BCIO:006103]:** A <self-regulation process> that modulates the frequency, rate or extent of one's performance of a behaviour.
15	**Norms:** The attitudes held and behaviours exhibited by other people within a social group.	**Social representation of a behaviour [BCIO:050779]:** A <cognitive representation> about a behaviour that is shared by members of a social group. **Group belief [BCIO:050669]:** A <social group attribute> in which a majority of members of a group have the belief. **Normative behaviour [BCIO:006095]:** An <individual human behaviour> that is commonly enacted by people that are part of a social environmental system. **Group descriptive behavioural norm [BCIO:050670]:** A <social group attribute> a behaviour is common within a social group. **Group evaluative behavioural norm [BCIO:050671]: A** <social group attribute> in which members of the group share an evaluative belief of a behaviour.
16	**Subjective norms:** One’s perceptions of what most other people within a social group believe and do.	Perceived norm [BCIO:006039] *: A <belief about one's social environment> in terms of what is typical for people who belong to a particular group. • **Perceived descriptive behavioural norm [BCIO:006040]**: A <perceived norm> regarding the prevalence of performance of a given behaviour by people within a group. • **Perceived evaluative behavioural norm [BCIO:006041]:** A <perceived norm> regarding whether a behaviour is appropriate and correct for people who belong to a particular group. • **Normative belief [BCIO:006042]:** A <perceived norm> regarding whether key others think one should perform a behaviour.
17	**Attitude towards the behaviour:** The general evaluations of the behaviour on a scale ranging from negative to positive.	**Evaluative belief about behaviour [BCIO:006147]:** An <evaluative belief> about whether a behaviour is positive or negative. **Affective attitude towards a behaviour [BCIO:050327]:** An <affective attitude> in which the entity that is the attitude object is a behaviour. **Attitude towards a behaviour [BCIO:050329]:** An <attitude> in which the entity that is the attitude object is a behaviour.
18	**Motivation:** Processes relating to the impetus that gives purpose or direction to behaviour and operates at a conscious or unconscious level.	Behavioural motivation [BCIO:006133] *: A <mental process> that energises and directs a behaviour. • **Automatic behavioural motivation [BCIO:006134]:** <Behavioural motivation> that arises from emotions and impulses that result from associative learning or innate dispositions. • **Reflective behavioural motivation [BCIO:050318]:** <Behavioural motivation> that involves reflective thinking.
19	**Self-image:** One’s conception and evaluation of oneself, including psychological and physical characteristics, qualities and skills.	**Evaluation of self [BCIO:006035]:** An <evaluative belief> about one's attributes. **Self-identity [ADDICTO:0000399]:** An <identity> that a person has about themselves.
20	**Needs:** Deficit of something required for survival, well-being or personal fulfilment.	**Psychological need [BCIO:006064]:** A <mental disposition> of a person to act to obtain or maintain a particular state due to this state’s importance to the person’s wellbeing. **Subjective need [BCIO:050316]:** A <subjective affective feeling> that is an attraction to an imagined scenario involving anticipated relief from or avoidance of mental or physical discomfort. **Physiological need [BCIO:050734]: A** <bodily disposition> resulting from a discrepancy between a current and target physiological state.
21	**Values:** Moral, social or aesthetic principles accepted by an individual or society as a guide to what is good, desirable or important.	**Personal value [BCIO:006063]:** A <mental disposition> to regard certain things as fundamentally important in life, which informs standards for behaviour.
22	**Feedback processes:** Processes through which current behaviour is compared against a particular standard.	**Social comparison process [BCIO:006118]: <**Judging> oneself or one's social group in relation to the qualities or characteristics of another person or social group. **Mentally comparing against a standard [BCIO:006132]:** A <mental process> in which conditions are compared against a particular reference level. **Feedback process to a person [BCIO:050663]:** A <process> in which information about a bodily process is received by the person.
23	**Social learning/imitation:** A process by which thoughts, feelings and motivational states observed in others are internalised and replicated without the need for conscious awareness.	Observational learning [GO:0098597] *: <Learning> that occurs through observing the behaviour of others. • **Imitative learning [GO:0098596]:** <Observational learning> in which new behaviours are acquired through imitation. • **Vicarious learning [BCIO:050794]:** <Observational learning> through the feelings or actions of another person.
24	**Behavioural cueing:** Processes by which behaviour is triggered from either the external environment, the performance of another behaviour, or from ideas appearing in consciousness.	**Behavioural cue [BCIO:050578]:** A <stimulus> that prompts a behaviour or a behaviour pattern. **Reinforcer [BCIO:050756]:** A <stimulus> that changes the likelihood of a preceding behaviour.
25	**General attitudes/beliefs:** Evaluations of an object, person, group, issue or concept on a scale ranging from negative to positive.	**Evaluative belief [BCIO:006038]:** A <belief> about whether a particular aspect of the world is positive or negative. **Affective attitude [BCIO:050326]:** A <mental disposition> to experience a subjective affective feeling about something. **Attitude [BCIO:050328]:** A <mental disposition> that is an affective attitude or an evaluative belief about something.
26	**Perceived susceptibility/vulnerability:** Perceptions of the likelihood that one is vulnerable to a threat.	**Belief about threat [BCIO:006306]:** A <belief> about a potential harm. **Belief about severity of an outcome [BCIO:006030]:** A <belief> about how serious the harm associated with an outcome could be. **Belief about susceptibility to a threat [BCIO:006305]:** A <belief> about vulnerability to a threat.

*Note*. MoA = Mechanism of Action; MAO = Mechanisms of Action Ontology; TaTT = Theory and Technique Tool * In some cases, both a class and its subclasses are shown in the mapping. This was done, as the relevant subclasses were considered to capture important aspects of an MoA group and would, therefore, be useful to view in the mapping.

Not all relevant subclasses are presented in this table, unless they capture important aspects of a TaTT MoA. Therefore, further engaging with the mapped ontology classes (e.g., viewing their subclasses) can help identify more detailed MoAs that are investigated or explored in studies. For example, the subclasses of “Memory process” [BCIO:050319] include: “Associative memory” [BCIO:006126], “Episodic memory” [BCIO:006127], “Iconic memory" [BCIO:006130], “Procedural memory” [BCIO:006129] and “Semantic memory” [BCIO:006128].

For reference, the earlier mapping of the MoA Ontology (released May, 2024) to the TaTT can be found here: https://osf.io/zmub5 and the initial mapping by the researchers: https://osf.io/ycdzv). During the coding process, some disagreements arose over how strictly ontology classes should be mapped to the 26 MoAs, given that the MoA Ontology contains more detailed and specific classes. These disagreements were resolved through discussions, and minor changes were made to the MoA Ontology where needed. Three classes were added to the MoA Ontology, with one class (e.g., “Affective attitude towards a behaviour” [BCIO:050327]) being added to more fully capture the MoA group “Attitude towards a behaviour” and two (“Attitude” [BCIO:050328] and “Affective attitude” [BCIO:050326]) to better capture “General Beliefs/Attitude”.

## Discussion

The purpose of this study was to align the TaTT and BCIO so that they can be used in combination. This was achieved by mapping the classes from the BCTO onto their corresponding BCTs in TaTT, and the classes from the MoA Ontology to their corresponding MoAs in TaTT. This mapping serves as a resource to develop interventions and more precisely report their BCT-MoA links, thereby helping build a stronger evidence base on the hypothesised pathways through which interventions change behaviour and identify gaps in research.

The current mapping, similar to the TaTT more generally, needs to be applied flexibly and considering evidence about target behaviours and their contexts ( Connell *et al*., 2019). BCT and MoA links greatly vary for interventions with different forms of delivery, schedules, levels of engagement, as well as for different target behaviours, populations and their settings ( Davidson & Scholz, 2020; Michie *et al*., 2020; Perski *et al*., 2017). Therefore, intervention developers need to take this variation into account, when identifying MoAs and selecting appropriate BCTs using the TaTT and the associated mapping to ontologies. Details about aspects of interventions can be reported using other BCIO ontologies for: intervention mode of delivery ( Marques *et al*., 2021), source ( Norris *et al*., 2021), schedule ( Marques *et al*., 2024a), engagement, setting ( Norris *et al*., 2020), population ( Wright *et al*., 2025) and target behaviour ( Schenk *et al*., 2024a).

An advantage of both the TaTT and BCIO is that they are tools that can be improved through the feedback from users and the wider behaviour change community ( Johnston *et al*., 2021; Michie *et al*., 2020; National Academies of Sciences, 2022). Up-to-date evidence about BCT-MoA links from the wider community can help improve the TaTT, making its mapping more nuanced with reference to relevant papers or databases ( Johnston *et al*., 2021). Similarly, feedback to the BCIO (e.g., regarding missing classes or definitions that need to be clarified) help these ontologies become more usable and widely applicable. This can be done by creating a “New Issue” on the ontology’s GitHub ( https://github.com/HumanBehaviourChangeProject/ontologies/issues). However, the potential for improving these tools is contingent on the behaviour change community actively using and critically engaging with them.

### Use of the mapping between the BCIO and TaTT

Intervention developers may use the TaTT as a starting point for identifying links between BCTs and MoAs. After verifying these links are relevant for their target behaviour and specific context (e.g., through a literature search or stakeholder consultations), the BCTO and MoA Ontology can be used to identify more granular classes. This helps intervention developers and evaluators specify and investigate what specific MoA a BCT targets, providing clearer guidance. For example, a BCT can be linked more specifically to the MoA “belief about the positive social consequences” (BCIO:050608) instead of the more general MoA “belief about consequences” MoA. These classes, with their computer-readable IDs (URIs), can then be used when reporting the hypothesised BCT-MoA links in protocols and papers, facilitating study replication and the accumulation of evidence. An example workflow of using the TaTT alongside the BCIO to guide the intervention development is presented in Table 4.

**Table 4.** Example workflow of using the TaTT, alongside BCIO mapping, during intervention development.

**What is the behaviour that needs to change?**	*Example: Adherence to physical distancing during Covid-19*
**What MoA(s) could be targeted to change the behaviour?**	*Example: remembering to maintain social distancing* • *In the TaTT, this MoA corresponds to “Memory, Attention, Decision Processes”, which broadly captures several more different processes* • *The corresponding and relevant class in the MoA Ontology = Memory [BCIO:050319]*
**What BCTs might change the behaviour?**	*In the TaTT, suitable BCT links to the MoA are:* • *7.1. Prompts/cues* • *11.3. Conserving mental resources* *The corresponding classes in the BCT Ontology are:* • *Prompt intended action BCT [BCIO:007080]* • *Cue BCT [BCIO:007081]* • *Conserve mental resources BCT [BCIO:007134]*

To further illustrate the example presented in Table 4, intervention developers may identify “remembering to social distance” as a potential MoA for the target behaviour “adherence to physical distancing during Covid-19” in the UK. They may then use the TaTT, BCIO and the current mapping as follows:

- The developers map their MoA onto the TaTT MoAs, identifying **“Memory, Attention and Decision Processes”** as the relevant MoA grouping.
- From the TaTT-MoA Ontology mapping for “Memory, Attention and Decision Processes”, the developers identify the relevant class to capture remembering to social distance: **“Memory” [ *BCIO:050319]***, thereby excluding the class ***“Decision-making” [BCIO:006116]*** which is not relevant for the MoA of interest
- Using the TaTT, the developers identify the following BCTs as potential links for “Memory, Attention and Decision Processes”: “ **7.1. Prompts/cues”** and **“13.1. Conserving mental energy”**.
  - For this example, we will assume that a literature search helps narrow down the selection to the BCT **“7.1. Prompts/cues”** to target remembering to social distance.
- From the TaTT-BCTO mapping for “7.1. Prompts/cues”, the developers identify the corresponding and more precisely defined BCTs: “ ***Prompt intended action BCT” [BCIO:007080]*** and ***“*** **Cue BCT” [BCIO:007081].**
  - Based on the context and evidence, intervention designers select one of these BCTs, or where relevant, both. For the example, we will assume “ ***Prompt intended action BCT” [BCIO:007080]*** is more relevant.
- The identified BCTO and MoA Ontology classes (with their precise definitions and computer-readable IDs) are reported, alongside their TaTT counterparts, in the intervention development protocol and paper.

**Figure 6.**
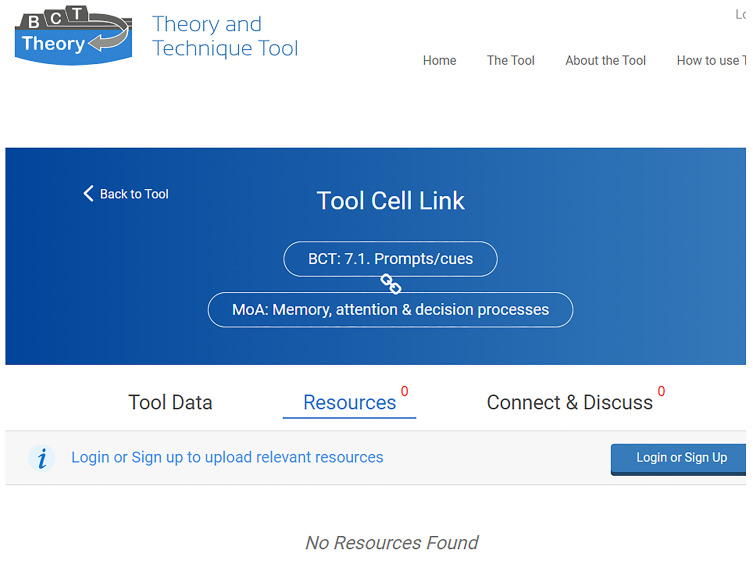
Screenshot of the Theory and Technique Tool (TaTT)’s resource section for a BCT-MoA link.

The developers may go on to evaluate their new intervention. Following this evaluation study, an additional step would be to provide feedback about a BCT-MoA link to the TaTT. This can be done by uploading the published paper to the relevant BCT-MoA link’s “Resource” section. For example, this section for the “7.1 Prompts/cues (BCT)” and “Memory, attention & decision processes (MoA)” can be found in the following link and is shown in Figure 6: https://theoryandtechniquetool.humanbehaviourchange.org/tool/1116/resources).

### Strengths and limitations

The current study supports better integration between the TaTT, which guides intervention development, and the BCIO, which supports precise reporting and evidence synthesis about behaviour change interventions. As the tools have been developed through different methods and for different purposes, the current work does not provide a one-to-one mapping between tools. Instead, users need to make judgements based on evidence when applying this mapping (e.g., to select more granular MoAs in the MoA Ontology). For the current mapping, our methods also relied on subjective judgements by researchers and consensus building among the wider research team. However, in the future, the mapping could be refined through feedback from TaTT and ontology users.

Beyond the links presented in the TaTT and this mapping, there are numerous additional links that could be proposed for the wider range of BCTs and MoAs in the ontologies. However, creating such a mapping between every BCT and MoA from the ontologies would be very time and resource intensive, and the resulting map is likely to be too detailed to be useable for practitioners. The current mapping provides a feasible way to engage with the more practical TaTT and the more detailed and precise ontologies. A final challenge in developing and maintaining the mapping is the need to update it whenever changes are made to the BCTO or MoA Ontology, as highlighted by current iterative methods.

### Future directions

The current study provides a starting point for extending the TaTT to incorporate BCTs and MoAs from the BCIO, as part of the 5-year NIH-funded project, The Advancing Prevention Research in Cancer through Ontology Tools (APRICOT) Project ( Michie *et al*., 2024). This project is developing a series of tools and resources to extend the uses of ontologies in the behavioural and social sciences and make them more accessible and useable ( Sharp *et al*., 2023). The APRICOT Project will help keep this mapping current over the project’s 5-year span, as well as develop the TaTT mapping using the BCIO to capture more detailed BCT-MoA links for specific target behaviours, such as physical activity.

Another area needing further development is the creation of improved measurements for MoAs ( Cornelius *et al*., 2024). This would allow us to test whether changes in specific MoAs, or combinations of them, actually bring about the effect of BCTs on behaviour. A previous study organised measures from a measurement repository by the Science of Behavior Change Network (SOBC; https://measures.scienceofbehaviorchange.org/) onto the 26 MoAs within the TaTT. ( Cornelius *et al*., 2023; Nielsen *et al*., 2018). More recent efforts have focused on mapping these measurements to the more precise MoA Ontology classes, offering a clearer view of which MoAs each measurement targets ( Cornelius *et al*., 2024; Schenk *et al*., under review). Since most measurements were linked to multiple MoAs, this work underscores the challenges in precisely measuring MoAs to whether interventions effectively modify specific MoAs to influence behaviour. To provide clearer guidance on how to test MoAs for each ‘likely’ BCT-MoA link, future work could attempt to: (1) collate and assess the quality of more precise measurements for specific MoAs, and/or (2) formally represent the combinations of classes from the MoA Ontology that measurements seem to assess.

## Conclusion

The current mapping serves as a starting point for the work to integrate TaTT and BCIO, as part of the APRICOT project. This will facilitate more evidence-based intervention design, and precise and computer-readable reporting of BCT- MoA links. The online platforms of the TaTT and BCIO will facilitate collaborative use and development of the tools. As these tools are used more widely and user feedback is integrated into them, they can increasingly contribute to a stronger evidence base on BCTs, MoAs, and their links.

## Ethics and consent

Ethical approval and consent were not required.

## Data Availability

No data associated. Open Science Framework: Human Behaviour-Change Project, https://doi.org/10.17605/OSF.IO/QRGC4 (West *et al.*, 2020). This project contains the following extended data: The BCTO released in May, 2024; https://osf.io/ya74q The previous mapping of the BCTO classes onto the TaT Project’s BCTs; https://osf.io/r7cux The MoA Ontology released in May, 2024; https://osf.io/pkq4e The previous mapping of the MoA Ontology classes onto the TaT Project’s MoA groups; https://osf.io/zmub5 OSF page for the Human Behaviour-Change Project; Homepage for all outputs across the project; https://osf.io/h4sdy/ Zenodo: HumanBehaviourChangeProject/ontologies: HumanBehaviourChangeProject/ontologies: Behaviour Change Technique Ontology, Mechanism of Action Ontology. https://doi.org/10.5281/zenodo.14882463 ( Schenk *et al*., 2025) Data are available under the terms of the Creative Commons Attribution 4.0 International license (CC-BY 4.0).

## Footnotes

^1^ A ‘likely’ BCT-MoA link refers to a link that (1) is explicitly hypothesised or identified in the literature and (2) agreed upon by behavioural experts to exist (i.e., that the BCT changes behaviour through the MoA), and was identified through the triangulation of evidence in the literature and an expert consensus study ( Johnston *et al*., 2018).

^2^ These 12 MoAs were identified by first judging which constructs from the 83 theories qualified as MoAs and then grouping the same and very similar MoAs together ( Schenk *et al*., 2024b). The most frequently occurring MoAs (i.e., the groups with the highest number of MoAs) were identified and compared to the 14 domains (potential MoAs) of the Theoretical Domains Framework ( Cane *et al*., 2012). The 12 most frequently occurring groups, which were not covered by the Theoretical Domains Framework, were included in the 26 MoAs for the TaTT. It should be noted that the complete list of theoretical constructs (from the 83 theories) also served as starting point for the MoA Ontology, but additional work was done to more precisely capture relevant constructs as MoAs and structure them within the ontology ( Schenk *et al*., 2024b)
