# Supplementary material for: Linking behaviour change techniques to mechanisms of action: Using the Theory and Techniques Tool alongside the Behaviour Change Intervention Ontology
Source: Wellcome Open Res. 2025 Oct 23;10:192. Originally published 2025 Apr 11. [Version 2] doi: 10.12688/wellcomeopenres.23879.2 (PMC12596556; doi:10.12688/wellcomeopenres.23879.2)
Supplement: Peer review history

# Reviewer report: Reviewer response for version 2

**DOI:** 10.21956/wellcomeopenres.27596.r137278

**Published:** 2025-11-18

**Authors:** Chris Keyworth

**Affiliations:** 1 University of Leeds, Leeds, UK

Thank you for the opportunity to review this manuscript on linking behaviour change techniques and mechanisms of action by aligning the Theory and Techniques Tool with the BCIO. This is an interesting and very valuable approach that will be extremely useful for researchers and practitioners working in intervention development and evaluation. I have provided some comments below, which I hope are helpful.

**General Comments**

 This study aimed to map the newer ontology categories to the TaTT’s BCTs and MoAs, thereby creating an alignment between the TaTT and BCIO for better integration in intervention design and evaluation.

**Unmapped BCTs - **The paper notes that not all BCTs in the newer ontology could be mapped to the TaTT BCTs. Would it be helpful to clarify what this means in practice. Should these unmapped BCTs be disregarded? Are they considered outside the TaTT’s scope? Some explanation of how users should interpret or handle these classes would strengthen the practical guidance.

**Examples of MoAs - **Where the manuscript states that the mapping supports intervention designers to “select, target and test their interventions’ MoAs (e.g., motivation, capability, opportunity),” it may be helpful to include the some examples, such as those provided in the abstract e.g. skill development or access to resources, to make this more concrete.

**Specific comments (mostly methodological)**

 p. 8

 “For a class to be considered captured by a TaTT BCT, it needed to either (1) have a definition with the same meaning… or (2) include all the attributes…”

 This is quite a specific coding rule. Was this definition from existing literature, or was it developed by the research team for this study?

 p. 9

 “Four new classes were added.”

 Does this refer to classes of BCTs?

 p. 10

 “For a class to be considered as captured by an MoA…”

 Similarly, it would be helpful to explain whether this coding rule was informed by prior research or developed internally by the team.

 p. 10

 “This approach differed from how we mapped BCTs…”

 I found this a little difficult to follow. Including one or two brief examples here, as you do in the subsequent sentences, would make the distinction clearer.

 Reliability / agreement - The manuscript reports that independent coding was conducted and disagreements were resolved through discussion. Were any coder agreement metrics calculated? Formal reliability statistics may not be essential for this type of conceptual mapping, if any initial coder agreement (e.g., percentage overlap) was caried out it might be useful to document.

 p. 19

 “Some disagreements arose…”

 A brief indication of the general nature of these disagreements (e.g., conceptual boundaries, hierarchical placement) would help readers understand the complexity of the task.

 Regarding “Minor changes were made to the MoA Ontology… Three classes were added…” - It may be useful to explicitly state whether these three added classes constitute all the “minor changes” referenced, and briefly summarise the rationale for each addition, to make the revision process more transparent.

Is the work clearly and accurately presented and does it cite the current literature?

Yes

If applicable, is the statistical analysis and its interpretation appropriate?

Yes

Are all the source data underlying the results available to ensure full reproducibility?

Yes

Is the study design appropriate and is the work technically sound?

Yes

Are the conclusions drawn adequately supported by the results?

Yes

Are sufficient details of methods and analysis provided to allow replication by others?

Yes

Reviewer Expertise:

Health psychology / behavioural science

I confirm that I have read this submission and believe that I have an appropriate level of expertise to confirm that it is of an acceptable scientific standard.

# Reviewer report: Reviewer response for version 2

**DOI:** 10.21956/wellcomeopenres.27596.r137027

**Published:** 2025-11-08

**Authors:** Mor Peleg

**Affiliations:** 1 University of Haifa, Haifa, Israel

I thank the authors for their thoughtful and elaborate response.

 I understand now that the aim was not to formalise the TaTT using ontological expressions. The TaTT is used in practical contexts, often for intervention development, or for understanding interventions theoretically. Accordingly, the mapping to the BCIO is intended to help behavioural and social scientists report BCTs and MoAs more precisely and formally, where relevant, particularly in protocols and papers about behaviour change interventions.

 Based on this explanation, almost all of my comments were resolved.

 26, 28. Thank you for making the change – it is now resolved

 Only one outstanding comment remains, related to my previous Comment #2. I suggested that the author add the following sentence (copied from their response letter) to the paper itself: "In order to make TaTT a practical tool that is useful to a wide range of people studying behaviour, the authors did not use formal ontological language in describing BCTs or MoAs."

Is the work clearly and accurately presented and does it cite the current literature?

Yes

If applicable, is the statistical analysis and its interpretation appropriate?

Not applicable

Are all the source data underlying the results available to ensure full reproducibility?

Yes

Is the study design appropriate and is the work technically sound?

Partly

Are the conclusions drawn adequately supported by the results?

Partly

Are sufficient details of methods and analysis provided to allow replication by others?

Yes

Reviewer Expertise:

biomedical ontologies, knowledge representation, mobile health, computer-interpretable clinical guidelines, clinical decision-support systems

I confirm that I have read this submission and believe that I have an appropriate level of expertise to confirm that it is of an acceptable scientific standard.

# Reviewer report: Reviewer response for version 1

**DOI:** 10.21956/wellcomeopenres.26345.r122374

**Published:** 2025-05-28

**Authors:** Elin M Andersson

**Affiliations:** 1 Department of Public Health and Clinical Medicine, Umea University, Umeå, Sweden

To aid researchers and practitioners in selecting behaviour change techniques (BCTs) that effectively target specific mechanisms of actions (MoAs), the **Theory and Technique Tool (TaTT)** was previously developed. This tool offers a matrix indicating which BCTs are likely—or unlikely—to influence particular MoAs. More recently, as part of The Behaviour Change Intervention Ontology, the two ontologies of the **Behaviour Change Technique Ontology (BCTO)** and the **Mechanisms of Action Ontology (MoA Ontology) **have been introduced. These ontologies expand the range of BCTs and MoAs, offering more precise, standardised, and machine-readable definitions.

 The aim of this study was to enhance the utility of the TaTT by aligning it with the above-mentioned ontologies. Potentially, this could:

1. Enabling ontology users to formulate hypotheses about likely BCT–MoA relationships.
2. Helping TaTT users to identify more detailed and relevant BCTs and MoAs for intervention design and research.

Introduction: The introduction provides a summary of previous work of relevance for this study, and outline the development of the TaTT, the BCTO and the MoA. I find that the level of detail is well-balanced; the text gives an overview and is clear and easy to follow, and the relevant references describing this rigorous work more in detail is included. I also believe the included figures should be helpful for readers without previous experience of the TaTT.

 Method: The steps taken are clearly described. However, I struggled with this sentence: “Unlike the BCT mapping, the researchers did not record multiple different TaTT MoAs for a single class from the MoA Ontology.” To me, the formulation is confusing, but the example that follows clarifies.

 Results: The tables are easy to read and search through. When this is further developed, perhaps the Tool Cell Link in the TaTT, (including the chosen specified BCT and Moa) could be expanded to also include the corresponding BCTO- and MoA classes? I realize that this would make the website more difficult to read, but perhaps a solution would be to make the BCT and the MoA in the Tool Cell Link clickable (so that the ontology classes are shown only for users who wish to see them). I believe that the suggested workflow of using the TaTT alongside BCIO mapping during intervention (presented in the discussion) could be facilitated if the link to the ontologies is built into the TaTT. Perhaps this is already planned?

 Discussion: The discussion includes several important considerations. The importance of that the mapping is applied with flexibility, considering evidence about target behaviours, populations and the context; Furthermore, that the potential for improving these tools is contingent on the behaviour change community actively using and critically engaging with them, including also to provide feedback about BCT-MoA-links; The need of improved measurements for MoA is also pointed out.

 I appreciate the opportunity to review this study, not least since I, alongside other colleagues, hoped for that the TaTT would be updated when the BCIO was launched. This study is definitely an important starting point for the work of integrating the TaTT and BCIO.

Is the work clearly and accurately presented and does it cite the current literature?

Yes

If applicable, is the statistical analysis and its interpretation appropriate?

Not applicable

Are all the source data underlying the results available to ensure full reproducibility?

Yes

Is the study design appropriate and is the work technically sound?

Yes

Are the conclusions drawn adequately supported by the results?

Yes

Are sufficient details of methods and analysis provided to allow replication by others?

Yes

Reviewer Expertise:

Health psychology; CVD prevention; Lifestyle modification; Risk communication; Efficacy beliefs

I confirm that I have read this submission and believe that I have an appropriate level of expertise to confirm that it is of an acceptable scientific standard.

# Response

**Published:** 2025-09-26

**Authors:** Paulina Margarete Schenk

**Affiliations:** University College London, London, England, UK

**Reviewer 2:** *To aid researchers and practitioners in selecting behaviour change techniques (BCTs) that effectively target specific mechanisms of actions (MoAs), the **Theory and Technique Tool (TaTT)** was previously developed. This tool offers a matrix indicating which BCTs are likely—or unlikely—to influence particular MoAs. More recently, as part of The Behaviour Change Intervention Ontology, the two ontologies of the **Behaviour Change Technique Ontology (BCTO)** and the **Mechanisms of Action Ontology (MoA Ontology) **have been introduced. These ontologies expand the range of BCTs and MoAs, offering more precise, standardised, and machine-readable definitions.*

*The aim of this study was to enhance the utility of the TaTT by aligning it with the above-mentioned ontologies. Potentially, this could: *

1. *Enabling ontology users to formulate hypotheses about likely BCT–MoA relationships.*
2. *Helping TaTT users to identify more detailed and relevant BCTs and MoAs for intervention design and research.*

**Response:** We thank the reviewer for engaging with this work and providing feedback on this manuscript.  

**Reviewer 2:** *Introduction: The introduction provides a summary of previous work of relevance for this study, and outline the development of the TaTT, the BCTO and the MoA. I find that the level of detail is well-balanced; the text gives an overview and is clear and easy to follow, and the relevant references describing this rigorous work more in detail is included. I also believe the included figures should be helpful for readers without previous experience of the TaTT.* **Response:** We thank the reviewer for their positive feedback on the Introduction.

**Reviewer 2:** *Method: The steps taken are clearly described. However, I struggled with this sentence: “Unlike the BCT mapping, the researchers did not record multiple different TaTT MoAs for a single class from the MoA Ontology.” To me, the formulation is confusing, but the example that follows clarifies.*

**Response:** We thank the reviewer for their feedback. The relevant passage has been updated to improve clarity:

- “ *The researchers did not do the reverse mapping, i.e., mapping TaTT MoAs to specific classes in the MoA Ontology. This approach differed from how we mapped BCTs, where it sometimes made sense to group several TaTT BCTs under broader classes in the BCTO. We avoided this here, because the TaTT MoAs are often very broad and can include subcomponents that belong to different parts of the MoA Ontology’s hierarchy. As a result, many TaTT MoAs would only map onto high-level structural classes (e.g., “mental disposition”), which are too broad to be practically meaningful. For example, the TaTT MoA “Social/Professional role & identity” includes the MoAs “personal role” [BCIO:006081] and “identity” [ADDICTO:0000381]. Since these are not closely related in the ontology’s logical hierarchy, the only possible mapping would be to a very general class such as “characteristic” [BFO:0000020] in the BCIO.* Therefore, to be more useable, the mapping between these tools was kept simpler.”

**Reviewer 2:** *Results: The tables are easy to read and search through. When this is further developed, perhaps the Tool Cell Link in the TaTT, (including the chosen specified BCT and Moa) could be expanded to also include the corresponding BCTO- and MoA classes? I realize that this would make the website more difficult to read, but perhaps a solution would be to make the BCT and the MoA in the Tool Cell Link clickable (so that the ontology classes are shown only for users who wish to see them). I believe that the suggested workflow of using the TaTT alongside BCIO mapping during intervention (presented in the discussion) could be facilitated if the link to the ontologies is built into the TaTT. Perhaps this is already planned?*

**Response:** We thank the reviewer for their suggestion. We are internally considering how best to integrate the results in a user-friendly way into the TaTT user interface.

**Reviewer 2:** *The current suggestion will be shared with the wider team and considered with the website developers to signpost the relevant classes from the ontologies for each BCT and MoA in the TaTT. Discussion: The discussion includes several important considerations. The importance of that the mapping is applied with flexibility, considering evidence about target behaviours, populations and the context; Furthermore, that the potential for improving these tools is contingent on the behaviour change community actively using and critically engaging with them, including also to provide feedback about BCT-MoA-links; The need of improved measurements for MoA is also pointed out.*

**Response:** We thank the reviewer for their positive feedback on the Discussion.

**Reviewer 2:** *I appreciate the opportunity to review this study, not least since I, alongside other colleagues, hoped for that the TaTT would be updated when the BCIO was launched. This study is definitely an important starting point for the work of integrating the TaTT and BCIO* **Response:** We thank the reviewer for their interest in the BCIO and the TaTT and their help in improving this paper.

# Reviewer report: Reviewer response for version 1

**DOI:** 10.21956/wellcomeopenres.26345.r122365

**Published:** 2025-04-29

**Authors:** Mor Peleg

**Affiliations:** 1 University of Haifa, Haifa, Israel

Many studies use behavior-change techniques (BCT) to facilitate behavior change. To allow the scientific community to compare different tools and systems related to behavior change, it is vital that studies relate to theories of behavior in a standard way; scientists should report the systems and their evaluation studies by referring to a standardized set of BCTs and the mechanisms of action (MoA) through which interventions bring about their influence on behaviour) to understand causal processes behind interventions. Two knowledge sources/tools have been created to facilitate such standardization: the TaTT and the BCI Ontology (BCIO). These resources conceptualize complementary and overlapping knowledge, yet they have not been mapped before. By mapping them, developers of BC Interventions could be facilitated by using the TaTT to  and then using the mappings to the BCIO to hypothesise potential links between the extended number of BCTs and MoAs found in the BCIO. This could allow researchers to identify and report the more detailed, clearly defined ontology classes in their protocols and papers and could link the papers to the TaTT.

 Detailed comments:

 Clarity

 1) "Clicking on any cell reveals the evidence for the relevant link from the three studies".

 I tried to click on the green cell that is discussed on p. 6: link between BCT “1.2 Problem Solving” and the MoA “BaCa”, which stands for the “Belief about Capabilities".

 I was expecting to see an explanation about the evidence. Instead I saw just metadata:

 - Triangulation study: study not required

 I was expecting to see some explanation why this link exists. Instead, I had to manually follow a link to the 2 studies (literature study and expert panel). Then to search for this link there. I saw that the expert study did not include any explanations for why they think the links exist. As a person trained in informatics and not in psychology, it would have been very helpful to me, if the TaTT would provide an explanation when I clicked on a green square for why links exists. I don't consider the fact that the studies found such links as an explanation. An explanation could cite from the literature study the basis for the link. However, the literature study also did not provide such an example. It would be useful to add examples to the TATT for green cells. For the example above, you could add that BCT1.2 could help a user solve a problem that she has related to barriers that she perceives to performing the behavior via the MoA of change in the belief about capabilities.

 2) Please note though, that I don't understand why in the TaTT, belief about capabilities is a MoA. It is a belief, a disposition, and this is correctly reflected in the BCIO as such. The MoA should be "change in belief".

 3) " by starting off with a TaTT MoA (e.g., “Memory, attention and decision process”) and then identifying the corresponding detailed ontology classes (e.g., “memory process” and “attending”), TaTT users can report more nuanced and varied evidence about BCT-MoA links or lack thereof"

 >> I don't understand how you would create an ontological expression that ties both ontology classes in a logical way. I also think that the part about decision process is not there. As in the comment above, memory_process and attending are both mental processes; they are not MoA. I guess that you meant that an expression should be created  "Mechanism_of_action_through_bodily_process" and 'through' some (memory_process OR mental_process)? On the other hand you say that users of TaTT find the ontology difficult to use and prefer to use TaTT. So I don't understand what value they would get from the ontology if they don't use the ontology for writing ontological expressions. What value would they get if they would know that some of the related classes in the ontology are Attending and Memory_process?

 Methods

 4) "In cases where a class did not align with a single TaTT BCT/MoA, the researchers recorded multiple TaTT BCTs/MoAs for the class, as needed."

 >>In my opinion it is not enough to just list classes that are needed to be composed; a class expression needs to be formulated by composing the classes via logical descriptions that are syntactically and semantically correct.

 I recommend including another step in the methods, which is the coding of the description logics class expressions in the ontology that convey the meaning of the TaTT MoAs and BCTs.

 In the comments below I used the structure of 'Class-I' 'Class-J' as a shorthand notation for expressions of the form:

 Class-I and relationship some Class-J

 5) "Unlike the BCT mapping, the researchers did not record multiple different TaTT MoAs for a single class from the MoA Ontology".

 > I don't understand: did you mean that there could be a 1:1 or a 1:N but not N:1 or N:M relations between TaTT MoAs and ontology-MoA class?

 6) I suggest adding annotation properties to the BCT and MoA classes in the ontologies that state which TaTT class is mapped to the BCT or MoA class. This should be true for 1:1 mappings but also for class descriptions added to represent complex MoAs.

 Results

 7) I think that mechanism of action is a process rather than a disposition. Many (all?) of your mappings from TaTT MoA are to (ontological) dispositions. Instead, I think that the modeling should be: mechanism of action (process) can be through a bodily disposition.

 8)  I think that the mapping of TaTT 'Social/Professional role & identity' should be represented in the following way:

 ‘mechanism of action through personal role’ ‘personal role’

 OR

 'mechanism of action through cognitive representation' ‘self-identity'

 9) Similarly for belief, use 'mechanism of action through bodily disposition' belief

 10) I don't understand why in the MoA, self-efficacy belief for a behaviour and its associated outcomes is not a subclass of self-efficacy belief for a behaviour

 I suggest the following hierarchy (note lines 2 and 3 below)

 self-efficacy belief

     self-efficacy belief for a behaviour

           self-efficacy belief for a behaviour and its associated outcomes

           situational self-efficacy belief for a behaviour

                self-efficacy belief for a behaviour in the face of social pressure

                self-efficacy belief for a behaviour under conditions of stress

          behavioural recovery self-efficacy

          behaviour maintenance self-efficacy

          self-efficacy belief for a behaviour during routine activities

    self-efficacy belief for avoiding a threat

 11) I disagree with the mapping of the Optimism TaTT MoA to the following two MoA ontology classes:

 Belief about likelihood of consequences of an occurrence [BCIO:006026]

 >> here there is nothing about a positive consequence

 Evaluative belief [BCIO:006038]: A about whether a particular aspect of the world is positive or negative.

 >> Perhaps there is a way to formulate a class expression that captures the correct mapping by referring to these 2 classes and to the value "positive". Also, to be a mechanism of action, I think that you should use the process "evaluative belief formation about a behaviour" BCIO:050650 rather than use belief, which is a disposition

 12) Reinforcement (TaTT)

 'mechanism of action through reinforcement process' 'reinforcement process'

 With a similar structure you can represent:

 'mechanism of action through bodily process' 'Internal reward for a response'

 However, this has an additional semantics compared to the TaTT reinforcement so please justify.

 13) Intention [TaTT]:

 'mechanism of action through bodily disposition' 'Behavioural intention'

 14) Goals [TaTT]:

 'mechanism of action through cognitive representation' Goal

 15) Memory, attention & decision processes

 >> See comment 3 above

 16) Environmental context & resources:

 'mechanism of action through environmental disposition' 'environmental disposition'

 I don't think that you need the Environmental System class, because the (textual) definition of 'environmental disposition' is: "A which is realised by an environmental system or system parts thereof"

 17) Social Influences

 'mechanism of action through bodily process' 'Socially-related behaviour'

 'mechanism of action through bodily process' 'Interpersonal process'

 18) Emotion

 'mechanism of action through bodily process'

 19) Behavioural regulation

 'mechanism of action through bodily process' Isn't this enough? Please justify why you also need:

 'mechanism of action through bodily disposition

 20) Norms

 'mechanism of action through bodily process' Why do you need Social representation of a behaviour? Given that 'normative behaviour'

  Is-a 'socially-related behaviour'?

 'mechanism of action through social group attribute' 'Group descriptive behavioural norm'

 'mechanism of action through social group attribute' 'Group evaluative behavioural norm'

 21) Subjective norms >> See 9 above

 22) Attitude towards the behaviour

 'mechanism of action through bodily disposition'   'Attitude' (or one of its subclasses)

 'mechanism of action through bodily disposition' 'evaluative belief '

 23) Motivation

 'mechanism of action through bodily process' 'Behavioural motivation'

 24) Self-image:

 Belief – see 9 above

 Identity – see 8 above

 25) Needs, Values

 >> as above, use with 'mechanism of action through bodily process'

 26) Feedback processes

 'mechanism of action through bodily process' 'Social comparison process'

 'mechanism of action through bodily process' 'Mentally comparing against a standard'

 **please explain how you could link 'Feedback process to a person' to one of the mechanism of actions of the ontology. I'm not sure how to do it.

 27) Social learning/imitation

 'mechanism of action through bodily process' 'Observational learning'

 28) Behavioural cueing

 Stimulus is a direct child of Entity in BCIO.

 **please explain how you could link it to one of the mechanism of actions of the ontology. I'm not sure how to do it.

 29) General attitudes/beliefs

 >> See above RE: beliefs, attitudes

 30) Perceived susceptibility/vulnerability

 >> See above RE: beliefs, attitudes

Is the work clearly and accurately presented and does it cite the current literature?

Yes

If applicable, is the statistical analysis and its interpretation appropriate?

Not applicable

Are all the source data underlying the results available to ensure full reproducibility?

Yes

Is the study design appropriate and is the work technically sound?

Partly

Are the conclusions drawn adequately supported by the results?

Partly

Are sufficient details of methods and analysis provided to allow replication by others?

Yes

Reviewer Expertise:

biomedical ontologies, knowledge representation, mobile health, computer-interpretable clinical guidelines, clinical decision-support systems

I confirm that I have read this submission and believe that I have an appropriate level of expertise to state that I do not consider it to be of an acceptable scientific standard, for reasons outlined above.

# Response

**Published:** 2025-09-26

**Authors:** Paulina Margarete Schenk

**Affiliations:** University College London, London, England, UK

**Reviewer 1:** *Many studies use behavior-change techniques (BCT) to facilitate behavior change. To allow the scientific community to compare different tools and systems related to behavior change, it is vital that studies relate to theories of behavior in a standard way; scientists should report the systems and their evaluation studies by referring to a standardized set of BCTs and the mechanisms of action (MoA) through which interventions bring about their influence on behaviour) to understand causal processes behind interventions. Two knowledge sources/tools have been created to facilitate such standardization: the TaTT and the BCI Ontology (BCIO). These resources conceptualize complementary and overlapping knowledge, yet they have not been mapped before. By mapping them, developers of BC Interventions could be facilitated by using the TaTT to  and then using the mappings to the BCIO to hypothesise potential links between the extended number of BCTs and MoAs found in the BCIO. This could allow researchers to identify and report the more detailed, clearly defined ontology classes in their protocols and papers and could link the papers to the TaTT.* **Response:** We thank the reviewer for engaging with the work in detail and appreciate their time in providing feedback to this paper.

**Reviewer 1 *:*** *Detailed comments:*

*Clarity*

*1) "Clicking on any cell reveals the evidence for the relevant link from the three studies".*

*I tried to click on the green cell that is discussed on p. 6: link between BCT “1.2 Problem Solving” and the MoA “BaCa”, which stands for the “Belief about Capabilities".*

*I was expecting to see an explanation about the evidence. Instead I saw just metadata:*

*- Triangulation study: study not required*

*I was expecting to see some explanation why this link exists. Instead, I had to manually follow a link to the 2 studies (literature study and expert panel). Then to search for this link there. I saw that the expert study did not include any explanations for why they think the links exist. As a person trained in informatics and not in psychology, it would have been very helpful to me, if the TaTT would provide an explanation when I clicked on a green square for why links exists. I don't consider the fact that the studies found such links as an explanation. An explanation could cite from the literature study the basis for the link. However, the literature study also did not provide such an example. It would be useful to add examples to the TATT for green cells. For the example above, you could add that BCT1.2 could help a user solve a problem that she has related to barriers that she perceives to performing the behavior via the MoA of change in the belief about capabilities.* **Response:** We thank the reviewer for their feedback on the level of detail included in the Theories and Techniques Tool (TaTT) regarding specific links. In line with the reviewer’s comments, the relevant sentence has been updated to be more precise in the manuscript’s Introduction (p. 6):

- *“Clicking on any cell reveals the meta-data about the relevant link from the three studies. However, for precise information on evidence regarding the links, the original studies should be referred to (* *Carey et al., 2019* *;* *Connell et al., 2019* *;* *Johnston et al., 2018,  2021* *).”*

We appreciate the feedback on the TaTT itself and will share this with the wider team. Details for what qualifies as a link within the expert consensus study is outlined by Connell et al., (2019), namely that 80% or more of participating experts needed to agree to a link. The TaTT did not set out to be explanatory, just to reflect the links between BCTs and MoAs that were hypothesised to be the case by experts as reported in the literature and in an expert consensus study. As the current work focused on mapping the TaTT and BCIO, updating the TaTT and how evidence is presented within it is beyond the scope of this work. 

**Reviewer 1:** *2) Please note though, that I don't understand why in the TaTT, belief about capabilities is a MoA. It is a belief, a disposition, and this is correctly reflected in the BCIO as such. The MoA should be "change in belief".* **Response:** We appreciate the reviewer’s comment. As suggested, in an intervention, a change in belief could constitute a mechanism of action (MoA). Here, ‘change’ also includes maintaining a belief at a level different from what it would have been without the intervention. The TaTT includes potential MoAs that can be influenced as part of an intervention. If an intervention successfully changes, for example, beliefs about capability, then (as the reviewer suggested) this change process would qualify as an MoA of that intervention. In behavioural science, it is useful to discuss constructs, such as beliefs about capability, as potential MoAs, since they represent targets for change. Particularly in the way that the TaTT is conceptualised, which links BCTs with ‘likely’ MoAs, there is an implication that constructs would be expected to change if they serve as the MoAs through which a BCT works. We have added the following clarification under the paragraph that introduces MoAs in the Introduction section:

- “ *While MoAs are processes through which interventions work to change behaviour, we often refer to states or dispositions (e.g., beliefs rather than changing or maintaining beliefs ) as MoAs in behavioural sciences. In this paper, we use similar language for brevity, but each time we refer to an MoA, it should be understood as either an MoA or a key construct that can be influenced and is part of an intervention’s MoA*.”

For clarity, the Introduction on the TaTT section has also been amended to be clearer and refer to ‘potential MoAs’:

- “ *To provide practical guidance on selecting BCTs to target **potential** MoAs in interventions, an online evidence-based grid that shows ‘likely’ BCT-MoA links* *^1^* *, the Theory and Technique Tool (TaTT **)**, was developed. These links were between 74 BCTs selected from the 93 BCTs of the Behaviour Change Techniques Taxonomy v1 (BCTTv1;* *Michie et al., 2013* *) and 26 **potential** MoAs. The 74 BCTs were the most commonly occurring ones, from the 93 BCTs, in a literature review (* *Carey et al., 2019* *).”*

The MoA Ontology’s upper level includes classes that formally represent “MoA through entity x.” Other high-level classes, such as “bodily disposition,” are related to classes explicitly formulated as MoAs (e.g., “MoA through bodily disposition”). In the paper describing the MoA Ontology, we explain that subclasses, such as “belief about capability”, are treated as MoAs through their hierarchical relationships. This approach avoids the need for a more complex hierarchy in which every class would be explicitly defined as “MoA through entity x” and separate classes would also be created for each “entity x.” However, we have now logged an issue on the GitHub issue tracker for the Behaviour Change Intervention Ontology on whether we will add ‘through’ relationships to each entity that qualifies as a potential MoA in the MoA Ontology (see https://github.com/HumanBehaviourChangeProject/ontologies/issues/1107). In order to make TaTT a practical tool that is useful to a wide range of people studying behaviour, the authors did not use formal ontological language in describing BCTs or MoAs. The MoA Ontology is more appropriate for contexts where formal language is required.

**Reviewer 1:** *3) " by starting off with a TaTT MoA (e.g., “Memory, attention and decision process”) and then identifying the corresponding detailed ontology classes (e.g., “memory process” and “attending”), TaTT users can report more nuanced and varied evidence about BCT-MoA links or lack thereof"*

*>> I don't understand how you would create an ontological expression that ties both ontology classes in a logical way. I also think that the part about decision process is not there. As in the comment above, memory_process and attending are both mental processes; they are not MoA. I guess that you meant that an expression should be created  "Mechanism_of_action_through_bodily_process" and 'through' some (memory_process OR mental_process)? On the other hand you say that users of TaTT find the ontology difficult to use and prefer to use TaTT. So I don't understand what value they would get from the ontology if they don't use the ontology for writing ontological expressions. What value would they get if they would know that some of the related classes in the ontology are Attending and Memory_process?* **Response:** We thank the reviewer for their comment on this. The feedback is on an example provided on how to use the BCIO (more specifically the MoA Ontology) to complement the TaTT when reporting a potential MoAs with more detail. To describe this example more precisely, the relevant sentence has been updated to read as follows:

- “ *For example, TaTT users could start off by identifying a TaTT MoA (e.g., “Memory, attention and decision process”) and then the corresponding detailed ontology classes for the potential MoA of interest (e.g., using the “memory process” class to specify the MoA more granularly where relevant). This would allow them to report more nuanced and varied evidence about BCT-MoA links or lack thereof.”*

Please note that, in the relevant extract and the current work more broadly, we did not aim to create ontological expressions for the TaTT MoAs (e.g., “Memory, attention and decision-making”) but provide an example of how the current mapping to the BCIO can be used for more precise reporting in conjunction with the TaTT.

**Reviewer 1:** *Methods*

*4) "In cases where a class did not align with a single TaTT BCT/MoA, the researchers recorded multiple TaTT BCTs/MoAs for the class, as needed."*

*>>In my opinion it is not enough to just list classes that are needed to be composed; a class expression needs to be formulated by composing the classes via logical descriptions that are syntactically and semantically correct.*

*I recommend including another step in the methods, which is the coding of the description logics class expressions in the ontology that convey the meaning of the TaTT MoAs and BCTs.*

*In the comments below I used the structure of 'Class-I' 'Class-J' as a shorthand notation for expressions of the form:*

*Class-I and relationship some Class-J* **Response:** Thank you for the suggestion. As the TaTT MoAs are very broad (e.g., “Memory, attention and decision-making”) and often include MoAs that do not share upper-level classes within the MoA Ontology, we suggest that the current mapping serves as a practical tool for using the BCIO and TaTT together, where appropriate. At this stage, the aim was not to formalise the TaTT using ontological expressions. The TaTT is used in practical contexts, often for intervention development, or for understanding interventions theoretically. Accordingly, the mapping is intended to help behavioural and social scientists report BCTs and MoAs more precisely and formally, where relevant, particularly in protocols and papers about behaviour change interventions. This can also be used to integrate evidence accumulated with both of these tools. 

**Reviewer 1:** *5) "Unlike the BCT mapping, the researchers did not record multiple different TaTT MoAs for a single class from the MoA Ontology".*

*> I don't understand: did you mean that there could be a 1:1 or a 1:N but not N:1 or N:M relations between TaTT MoAs and ontology-MoA class?* **Response:** Thank you for noting this; the description needed to be updated to be clearer. This extract, along with the broader explanation in the paragraph, aimed to convey that mapping the TaTT MoAs onto specific MoA Ontology classes would not for practical application. These TaTT MoAs are very broad and sometimes do not share a meaningful ‘parents’ in the MoA Ontology; therefore, they would be mapped onto very broad structural classes in the ontology. The relevant passage has been updated to improve clarity:

- “ *The researchers did not do the reverse mapping, i.e., mapping TaTT MoAs to specific classes in the MoA Ontology. This approach differed from how we mapped BCTs, where it sometimes made sense to group several TaTT BCTs under broader classes in the BCTO. We avoided this here, because the TaTT MoAs are often very broad and can include subcomponents that belong to different parts of the MoA Ontology’s hierarchy. As a result, many TaTT MoAs would only map onto high-level structural classes (e.g., “mental disposition”), which are too broad to be practically meaningful. For example, the TaTT MoA “Social/Professional role & identity” includes the MoAs “personal role” [BCIO:006081] and “identity” [ADDICTO:0000381]. Since these are not closely related in the ontology’s logical hierarchy, the only possible mapping would be to a very general class such as “characteristic” [BFO:0000020] in the BCIO. *Therefore, to be more useable, the mapping between these tools was kept simpler.”

**Reviewer 1:** *6) I suggest adding annotation properties to the BCT and MoA classes in the ontologies that state which TaTT class is mapped to the BCT or MoA class. This should be true for 1:1 mappings but also for class descriptions added to represent complex MoAs.*

**Response: **For the BCTs, the TaTT BCTs (with one-to-one mapping to the BCTO classes) are already cross referenced within the BCTO. This is now explicitly indicated in the manuscript:

- “The BCTs from the TaTT that have one-on-one mappings with BCTO classes are also cross-referenced within the ontology.”

As suggested, we have added the potential MoAs from the TaTT as relevant examples to the MoA Ontology when a one-to-one mapping exists (e.g., the example  “"Knowledge" from the Theory and Techniques Tool ( https://theoryandtechniquetool.humanbehaviourchange.org/tool)” has been added for the class “knowledge” [BCIO:006052] in the MoA Ontology). This is also indicated now in the manuscript:

- “ *The TaTT MoAs that have one-on-one mappings with classes in the MoA Ontology were explicitly included as examples in the ontology*.”

However, as indicate above, the mapping of very broad TaTT MoAs to single MoA Ontology is often not meaningful in practice and so these TaTT MoAs have not been added as examples to the ontology at this point. 

**Reviewer 1:** *Results*

*7) I think that mechanism of action is a process rather than a disposition. Many (all?) of your mappings from TaTT MoA are to (ontological) dispositions. Instead, I think that the modeling should be: mechanism of action (process) can be through a bodily disposition.* **Response:** As the reviewer suggested, mechanisms of action are processes, rather than dispositions. As also indicated above, the TaTT MoAs would only count as MoAs when they are part of a process through which the intervention works to influence behaviour. We have added the following clarification under the paragraph introducing MoAs in the Introduction:

- “ *While MoAs are processes through which interventions work to change behaviour, we often refer to states or dispositions (e.g., beliefs rather than changing or maintaining beliefs ) as MoAs in behavioural sciences. In this paper, we use similar language for brevity, but each time we refer to an MoA, it should be understood as either an MoA or a key construct that can be influenced and is part of an intervention’s MoA*.”

In the MoA Ontology, we include the formal structure “MoA through class x,” where class x refers to higher-level classes within the MoA Ontology. These upper-level MoA classes are formally linked to related classes. The idea is that lower-level classes are considered MoAs through this relationship and their hierarchical relationship. This approach was chosen during the development of the MoA Ontology to efficiently represent that MoAs can operate through various entities, including dispositions such as beliefs, without duplicating the hierarchy. We have now also raised an issue on GitHub to discuss whether we more precisely capture these lower-level classes by adding a ‘through’ relationship between them and the formal mechanism of action class (see https://github.com/HumanBehaviourChangeProject/ontologies/issues/1107). On the other hand, the TaTT is not an ontology and so does not follow the same structure. Potential MoAs or constructs that are part of MoAs are often referred to as MoAs within behavioural sciences. Within the TaTT, the influence on the constructs (e.g., belief about capability) is implied, as a BCT would be working through this construct (i.e., changing it) to influence behaviour. 

**Reviewer 1:** *8)  I think that the mapping of TaTT 'Social/Professional role & identity' should be represented in the following way:*

*‘mechanism of action through personal role’ ‘personal role’*

*OR*

*'mechanism of action through cognitive representation' ‘self-identity'* **Response:** We appreciate the recommendation. However, to avoid creating confusion for behavioural and social scientists, we would suggest keeping the mapping simpler, for example, only using the classes ‘personal role’ and ‘self-identity’. Within the MoA Ontology, these classes are linked to the relevant formal MoA class either directly or through their parent classes. As explained above, this approach can be used to capture MoAs more efficiently. The upper levels might be confusing to users who are unfamiliar with the formal language of ontologies and have limited time to engage with the MoA Ontology. However, as noted, we are considering adding the ‘through’ relationship to the more detailed classes in the MoA Ontology to make their link to the upper-level MoA class more explicit.

**Reviewer 1:** *9) Similarly for belief, use 'mechanism of action through bodily disposition' belief*

**Response:** As explained above, we would like to continue using the simple approach to capture these MoAs in our mapping. We opt for this approach to enable clearer communication with members of the behavioural and social sciences community. 

**Reviewer 1:**** *10) I don't understand why in the MoA, self-efficacy belief for a behaviour and its associated outcomes is not a subclass of self-efficacy belief for a behaviour*

*I suggest the following hierarchy (note lines 2 and 3 below)*

*self-efficacy belief*

*self-efficacy belief for a behaviour*

*self-efficacy belief for a behaviour and its associated outcomes*

*situational self-efficacy belief for a behaviour*

*self-efficacy belief for a behaviour in the face of social pressure*

*self-efficacy belief for a behaviour under conditions of stress*

*behavioural recovery self-efficacy*

*behaviour maintenance self-efficacy*

*self-efficacy belief for a behaviour during routine activities*

*self-efficacy belief for avoiding a threat*

**Response: **The class ‘self-efficacy belief about a behaviour and its associated behaviour’ has part ‘self-efficacy belief about a behaviour’ but is not strictly speaking a subclass of the latter. This relationship is indicated in the MoA Ontology. Please note that any feedback regarding the MoA Ontology and the structure of its classes can be submitted through the issue tracker on GitHub: https://github.com/HumanBehaviourChangeProject/ontologies/issues

**Reviewer 1:** *11) I disagree with the mapping of the Optimism TaTT MoA to the following two MoA ontology classes:*

*Belief about likelihood of consequences of an occurrence [BCIO:006026]*

*>> here there is nothing about a positive consequence*

*Evaluative belief [BCIO:006038]: A about whether a particular aspect of the world is positive or negative.*

*>> Perhaps there is a way to formulate a class expression that captures the correct mapping by referring to these 2 classes and to the value "positive". Also, to be a mechanism of action, I think that you should use the process "evaluative belief formation about a behaviour" BCIO:050650 rather than use belief, which is a disposition* **Response:** The definition of ‘Optimism’ (“Confidence that things will happen for the best or that desired goals will be attained”) captures the likelihood of an event and that this will be positive. However, the research team would recommend keeping the two classes mapped onto ‘Optimism’ broad as it can also involve beliefs that negative events are unlikely. We have added a clarification to the ‘Optimism’ that the combination of the two classes mapped to ‘Optimism’ are necessary to capture it:

- “ ** To capture ‘Optimism’ a combination of ‘belief about likelihood of consequences of an occurrence [BCIO:006026]’ and ‘evaluative belief [BCIO:006038]’ are needed, as optimism involves beliefs about likelihoods and an evaluative component. It should be noted that ‘Optimism’ can involve beliefs that negative events are unlikely.”

**Reviewer 1:** *12) Reinforcement (TaTT)*

*'mechanism of action through reinforcement process' 'reinforcement process'*

*With a similar structure you can represent:*

*'mechanism of action through bodily process' 'Internal reward for a response'*

*However, this has an additional semantics compared to the TaTT reinforcement so please justify.* **Response:** As explained above, we would like to continue using the simple approach to capture these MoAs in our mapping. We opt for this approach to enable clearer communication with members of the behavioural and social sciences community.

**Reviewer 1:**** *13) Intention [TaTT]:*

*'mechanism of action through bodily disposition' 'Behavioural intention*' **Response:** As explained above, we would like to continue using the simple approach to capture these MoAs in our mapping. We opt for this approach to enable clearer communication with members of the behavioural and social sciences community.

**Reviewer 1:** *14) Goals [TaTT]:*

*'mechanism of action through cognitive representation' Goal* **Response:** As explained above, we would like to continue using the simple approach to capture these MoAs in our mapping. We opt for this approach to enable clearer communication with members of the behavioural and social sciences community.

**Reviewer 1:** *15) Memory, attention & decision processes*

*>> See comment 3 above* **Response:** As explained above, we would like to continue using the simple approach to capture these MoAs in our mapping. We opt for this approach to enable clearer communication with members of the behavioural and social sciences community.

**Reviewer 1:** *16) Environmental context & resources:*

*'mechanism of action through environmental disposition' 'environmental disposition'*

*I don't think that you need the Environmental System class, because the (textual) definition of 'environmental disposition' is: "A which is realised by an environmental system or system parts thereof"* **Response:  **We agree that any MoA working through the environmental system is likely to relate to environmental dispositions (realised as processes). However, the MoA Ontology aimed to capture constructs that are often used in behavioural sciences, which includes both the environmental systems and dispositions or opportunities within these environments. Depending on how an MoA is conceptualised and operationalised in an intervention, it can involve the actual environment being changed (e.g., more walking pathways in the environment) or the disposition in the environment (e.g., the accessibility of an environment). While these are often overlapping and closely related, we lean towards representing both entities as classes for now. Regarding the second point of adding the higher-level classes in the MoA Ontology, please note the explanations provided above: We would like to continue using the simple approach to capturing these MoAs, as this can enable clearer communication with members of the behavioural and social sciences community.

**Reviewer 1:** *17) Social Influences*

*'mechanism of action through bodily process' 'Socially-related behaviour'*

*'mechanism of action through bodily process' 'Interpersonal process'* **Response:** As explained above, we would like to continue using the simple approach to capture these MoAs in our mapping. We opt for this approach to enable clearer communication with members of the behavioural and social sciences community.

**Reviewer 1:** *18) Emotion*

*'mechanism of action through bodily process'* **Response:** As explained above, we would like to continue using the simple approach to capture these MoAs in our mapping. We opt for this approach to enable clearer communication with members of the behavioural and social sciences community. 

**Reviewer 1:** *19) Behavioural regulation*

*'mechanism of action through bodily process' Isn't this enough? Please justify why you also need:*

*'mechanism of action through bodily disposition* **Response: **On the first note – adding the upper-level classes in the MoA Ontology – please note the explanations above about attempting to keep this mapping simple. The class ‘self-regulatory capability’ (a disposition) has been mapped to the TaTT ‘Behavioural regulation’ (definition: “Behavioural, cognitive and/or emotional skills for managing or changing behaviour.”), as the part of the definition on ‘skills’ would include capabilities. 

**Reviewer 1:** *20) Norms*

*'mechanism of action through bodily process' Why do you need Social representation of a behaviour? Given that 'normative behaviour'*

*Is-a 'socially-related behaviour'?*

*'mechanism of action through social group attribute' 'Group descriptive behavioural norm'*

*'mechanism of action through social group attribute' 'Group evaluative behavioural norm'*

**Response:** As norms can include representations or beliefs held by a group (‘Norms’ in the TaTT is defined as “The attitudes held and behaviours exhibited by other people within a social group”), we mapped the classes “social representation of a behaviour [BCIO:050779]” and “group belief [BCIO:050669]”, among others onto this TaTT MoA. On the second point, as explained above, we would like to continue using the simple approach to capture these MoAs in our mapping. We opt for this approach to enable clearer communication with members of the behavioural and social sciences community.

**Reviewer 1:** *21) Subjective norms >> See 9 above*

**Response:** As explained above, we would like to continue using the simple approach to capture these MoAs in our mapping. We opt for this approach to enable clearer communication with members of the behavioural and social sciences community.

**Reviewer 1:** *22) Attitude towards the behaviour*

*'mechanism of action through bodily disposition'   'Attitude' (or one of its subclasses)*

*'mechanism of action through bodily disposition' 'evaluative belief '* **Response:** As explained above, we would like to continue using the simple approach to capture these MoAs in our mapping. We opt for this approach to enable clearer communication with members of the behavioural and social sciences community. 

**Reviewer 1:**** *23) Motivation*

*'mechanism of action through bodily process' 'Behavioural motivation'* **Response:** As explained above, we would like to continue using the simple approach to capture these MoAs in our mapping. We opt for this approach to enable clearer communication with members of the behavioural and social sciences community.

**Reviewer 1:** *24) Self-image:*

*Belief – see 9 above*

*Identity – see 8 above* **Response:** As explained above, we would like to continue using the simple approach to capture these MoAs in our mapping. We opt for this approach to enable clearer communication with members of the behavioural and social sciences community.

**Reviewer 1:**** *25) Needs, Values*

*>> as above, use with 'mechanism of action through bodily process'* **Response:** As explained above, we would like to continue using the simple approach to capture these MoAs in our mapping. We opt for this approach to enable clearer communication with members of the behavioural and social sciences community.

**Reviewer 1:** *26) Feedback processes*

*'mechanism of action through bodily process' 'Social comparison process'*

*'mechanism of action through bodily process' 'Mentally comparing against a standard'*

**please explain how you could link 'Feedback process to a person' to one of the mechanism of actions of the ontology. I'm not sure how to do it. **Response:** We thank the reviewer for their comment, as they suggested an upper-level class was needed in the MoA Ontology to capture MoAs working through these feedback processes. This class has now been added to the ontology (“mechanism of action through feedback process to a person”). The number of classes in the MoA Ontology, reported in this manuscript, has also been updated to reflect the most recent release of the ontology. 

**Reviewer 1:** *27) Social learning/imitation*

*'mechanism of action through bodily process' 'Observational learning'* **Response:** As explained above, we would like to continue using the simple approach to capture these MoAs in our mapping. We opt for this approach to enable clearer communication with members of the behavioural and social sciences community.

**Reviewer 1:** *28) Behavioural cueing*

*Stimulus is a direct child of Entity in BCIO.*

**please explain how you could link it to one of the mechanism of actions of the ontology. I'm not sure how to do it. **Response:** We thank the reviewer for their comment, as they suggested an upper-level class was needed in the MoA Ontology to capture MoAs working through stimulus. This class has now been added to the ontology (“mechanism of action through stimulus”). The number of classes in the MoA Ontology, reported in this manuscript, has also been updated to reflect the most recent release of the ontology.

**Reviewer 1:** *29) General attitudes/beliefs*

*>> See above RE: beliefs, attitudes* **Response:** As explained above, we would like to continue using the simple approach to capture these MoAs in our mapping. We opt for this approach to enable clearer communication with members of the behavioural and social sciences community.

**Reviewer 1:** *30) Perceived susceptibility/vulnerability*

*>> See above RE: beliefs, attitudes* **Response:** As explained above, we would like to continue using the simple approach to capture these MoAs in our mapping. We opt for this approach to enable clearer communication with members of the behavioural and social sciences community. **References** Carey RN, Connell LE, Johnston M, et al.: Behavior Change Techniques and their Mechanisms of Action: a synthesis of links described in published intervention literature. *Ann Behav Med.* 2019;53(8):693–707. 30304386 10.1093/abm/kay078 6636886 Connell, L. E., Carey, R. N., De Bruin, M., Rothman, A. J., Johnston, M., Kelly, M. P., & Michie, S. (2019). Links between behavior change techniques and mechanisms of action: an expert consensus study. *Annals of behavioral medicine*, *53*(8), 708-720. Johnston M, Carey RN, Connell Bohlen L, et al.: Linking Behavior Change Techniques and Mechanisms of Action: Triangulation of findings from literature synthesis and expert consensus. *Ann Behav Med.* 2018. 10.31234/osf.io/ur6kz
